# Investigation of the Binding of the Macrolide Antibiotic Telithromycin to Human Serum Albumin by NMR Spectroscopy

**DOI:** 10.3390/ijms262412005

**Published:** 2025-12-13

**Authors:** Markus Rotzinger, Peter Hartmann, Barbara Muhry, Karina Stadler, A. Daniel Boese, Predrag Novak, Klaus Zangger

**Affiliations:** 1 Institute of Chemistry, University of Graz, Heinrichstraße 28, 8010 Graz, Austriapeter.hartmann@uni-graz.at (P.H.);; 2 Department of Chemistry, University of Zagreb, Horvatovac 102a, 10000 Zagreb, Croatia

**Keywords:** macrolide antibiotics, NMR, DOSY, transferred NOE, human serum albumin, DFT

## Abstract

The macrolide antibiotic telithromycin was developed to avoid common antibiotic resistances, yet it has been recently withdrawn from the European market due to severe side effects. Both side effects and the effectiveness of a drug can be related to the strength of its interaction with human serum albumin (HSA). However, as of yet, interactions between telithromycin and HSA have not been thoroughly studied. In this work, we evaluate the interaction strength and structural details of telithromycin and HSA via diffusion ordered spectroscopy (DOSY) and transferred NOE measurements. The binding strengths are compared with those of related macrolides. Our results show that the interaction strength increases with the decreasing polarity of the side chains in the antibiotic. Among the tested macrolide antibiotics, telithromycin interacted the strongest with HSA. Structure calculations based on transferred NOEs, using DFT calculations, show that telithromycin adopts a specific conformation upon binding, which shields the polar moieties attached to the aglycon and enables more hydrophobic interactions with HSA.

## 1. Introduction

Macrolides are a group of antibiotics that are used to treat bacterial infections against a series of pathogens. Among others, macrolides are particularly effective against *Legionella pneumophila*, *Chlamydia* spp., *Mycoplasma*, *Helicobacter pylori*, *Mycobacteria*, *Streptococci,* and methicillin-sensitive *Staphylococcus* [1]. This group of antibiotics inhibits bacterial growth by binding to the 23S rRNA component of the 50S ribosomal subunit, thus interrupting protein synthesis while simultaneously blocking ribosome assembly [2]. Erythromycin A was developed in 1952 and was the first macrolide antibiotic in clinical use. It consists of a 14-membered macrocyclic lactone ring with two sugar moieties: desosamine and cladinose. Since then, new structural derivatives of erythromycin were developed, exhibiting improved pharmacodynamic properties, such as longer serum half-life and better tissue penetration [3,4].

However, excessive use, misuse, and overdosing make macrolides susceptible to antibiotic resistance. Mutations in genes encoding for 50S ribosomal proteins, enzymatic inactivation by phosphotransferases or active efflux pumps, encoded by resistant strains, can severely diminish or cancel the antibiotic activity of macrolides [3,5]. Attempting to circumvent these problematic resistances led to a novel group of semi-synthetic derivatives of erythromycin, the ketolide antibiotics [6,7,8]. Telithromycin (TM), the first compound of this group available on the market, was designed by substitution of a keto group for a cladinose moiety and the addition of an N-substituted 11,12-carbamate side chain. Telithromycin maintains the favorable traits of conventional macrolides but shows a 10-fold higher affinity to the 50S ribosomal subunit compared to erythromycin. While older macrolides only show tight binding to domain V of the 23S rRNA component and only loose binding to other domains, telithromycin binds strongly with domains V and II, thus being able to bypass resistance mechanisms related to ribosomal binding. Moreover, telithromycin is also less prone to efflux mechanisms, rendering the development of resistance overall less likely for this antibiotic drug [3,8,9,10,11,12]. Ultimately, TM was withdrawn from the market due to studies indicating rare adverse effects like severe liver injury with liver failure [13], or neurological consequences like the exacerbation of myasthenia gravis [14].

Besides target site interactions, the efficacy of pharmaceutical compounds depends heavily on the degree of plasma protein binding, which has a strong impact on pharmacokinetic properties, such as clearance and half-life [15,16,17,18]. Moreover, drug binding to the plasma proteins such as human serum albumin (HSA), α-acid glycoprotein, and lipoproteins regulate the ratio of bound and unbound drug in the plasma. Stronger binding of a drug to plasma proteins results in a lowering of its free, active concentration and, concomitantly, an increased half-life. Usually, drugs bind to both HSA and α-acid glycoprotein, but with different affinities. While α-acid glycoprotein represents only 1–3% of total plasma proteins, HSA makes up about 60% (35–50 mg/mL). Due to a high diversity of available binding sites and conformational adaptability, HSA has an extraordinarily high binding capacity, which provides a depot for a large variety of ligands that may be available in quantities exceeding their solubility in plasma [19,20,21]. Erythromycin preferentially binds to α-acid glycoprotein and to a smaller extent to albumin (38–45% to HSA), with a total proportion of 70–90% plasma protein binding [22] (EMA 2000). On the contrary, TM is primarily bound to HSA with some involvement of α-acid glycoproteins and lipoproteins, with the total plasma protein binding reported to be around 60–90% [23,24,25].

Structurally, HSA is a 66 kDa, globular, monomeric protein with three homologous domains (I, II, III) that consist of 67% α-helices. Each domain consists of 10 helices and is divided into separate subdomains A and B, which are connected by loops. The protein carries nine binding sites for fatty acids, named FA1 to FA9. The main binding sites for pharmaceuticals are FA1, FA3–FA4, and FA7. FA1, also known as the heme binding pocket, is located in the center of subdomain IB. The cleft between FA3 and FA4 in subdomain IIIA, first described as Sudlow site II, forms a small hydrophobic pocket, mainly consisting of uncharged residues. Finally, FA7, originally called Sudlow site I, is considered the primary binding site of HSA. FA7 is located in subdomain IIA and includes three sub-sites, comprising charged residues to support hydrogen bonding [19,26,27,28].

The elucidation of the binding characteristics of pharmaceutical drugs to HSA is of high interest and has been investigated for a large variety of compounds. However, the drug binding sites, mechanisms, and affinities of erythromycin and telithromycin binding to HSA are not fully understood. Accurate predictions of plasma protein binding are crucial for the optimization of transport and substance release at the desired physiological site and are essential to avoid under- or overdosing related to free drug concentration [29,30].

**Figure 1:**
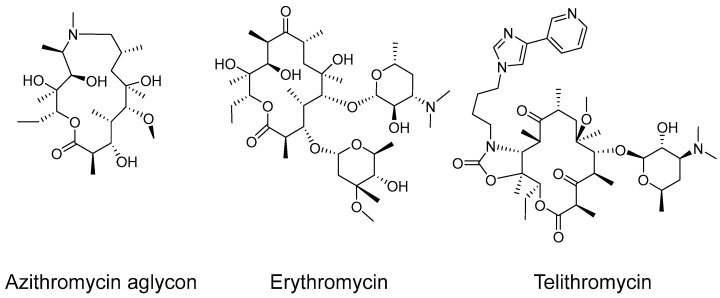
Structures of the macrolides used in this study.

In this work, we investigate the interaction between several representative macrolides (Figure 1) and HSA via nuclear magnetic resonance (NMR) spectroscopy, obtaining binding constants through diffusion ordered spectroscopy (DOSY). The change in the diffusion coefficient, which was obtained from 2D DOSY spectra in the absence and presence of the binding partner, correlates directly with the strength of the interaction [31]. The diffusion coefficients are evaluated comparatively and provide the mole fraction partition coefficient K_p_, which stands for the amount of ligand bound to the protein. This parameter allows for the direct comparison of the macrolide antibiotics to indicate the relative binding strength [32].

Furthermore, we were interested in whether TM shows a distinct binding conformation in its interaction with HSA. TM is known to occupy a large conformational space, particularly with respect to its flexible carbamate sidechain. This variability in conformation functions as an adaptation of the molecule to the chemical environment [33]. Saturation transfer difference NMR spectroscopy (STD) has previously been employed to study interactions of some macrolide antibiotics (azithromycin, oleandomycin, and telithromycin) with bovine serum albumin and revealed their binding epitopes [34]. Here we report the conformation of telithromycin bound to HSA, which was obtained using transferred NOE (trNOE)-derived distance restraints and structure calculations using forcefields and Density Functional Theory (DFT) [35,36,37].

## 2. Results and Discussion

**Figure 2:**
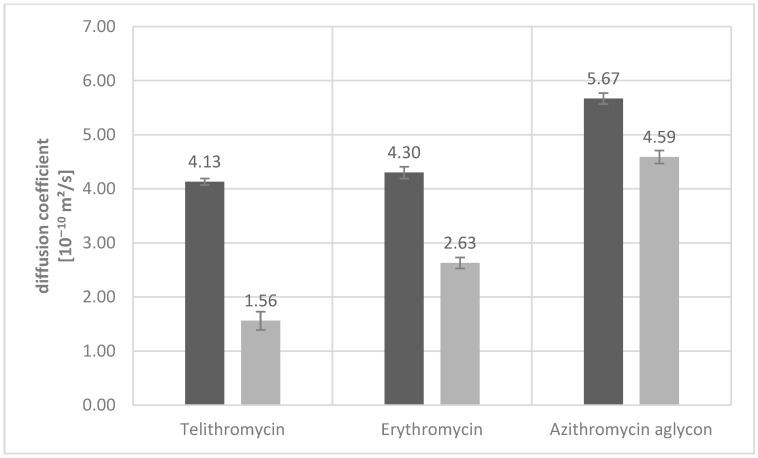
Self-diffusion coefficients of free macrolides (dark gray) and in the presence of HSA (light gray) at a ligand: protein ratio of 40:1. Ligand concentrations of 1 mM for erythromycin and azithromycin aglycon were chosen. The reduced concentration of 0.5 mM for telithromycin was chosen due to limited solubility in D_2_O. The diffusion coefficient of HSA was determined using the same parameters at a higher number of scans to be 0.67 ± 0.03·10^−10^ m^2^/s.

To deepen our insight into the pharmacokinetic behavior of macrolide antibiotics, we started out by investigating several representative members of this class regarding their interaction strengths with HSA. For this purpose, NMR spectroscopy lends itself as a potent tool to directly determine diffusion coefficients and detect interaction strengths. Accordingly, diffusion ordered spectroscopy (DOSY) spectra were recorded to determine the diffusion coefficients of the macrolides in the absence and presence of HSA, respectively (Figure 2). HSA offers several potential binding sites that can interact with different TM conformations. The evaluation of DOSY data yields a simplified representation of the interaction, because the observed change in diffusion coefficient reflects an average over different conformations at potentially different locations on HAS, all of which can influence the diffusion behavior slightly differently. Consequently, DOSY measurements can only provide a rough picture of the binding behavior of any ligand that can occupy different binding areas on a protein with different ligand conformations.

Expectedly, the compounds show an increase in their free diffusion coefficient (4.13 vs. 5.67 m^2^/s), congruent with the decrease in molecular weight (812 vs. 419 g/mol) from telithromycin to azithromycin aglycon. The increase is not directly proportional but is affected by the hydrophobicity of the side chains and their orientation, resulting in a non-spherical molecular shape. From the ratio of the macrolide in the absence and presence of the protein, the mole fraction partition coefficients (K_p_) can be calculated, giving information on the relative binding strengths to HSA.

**Figure 3:**
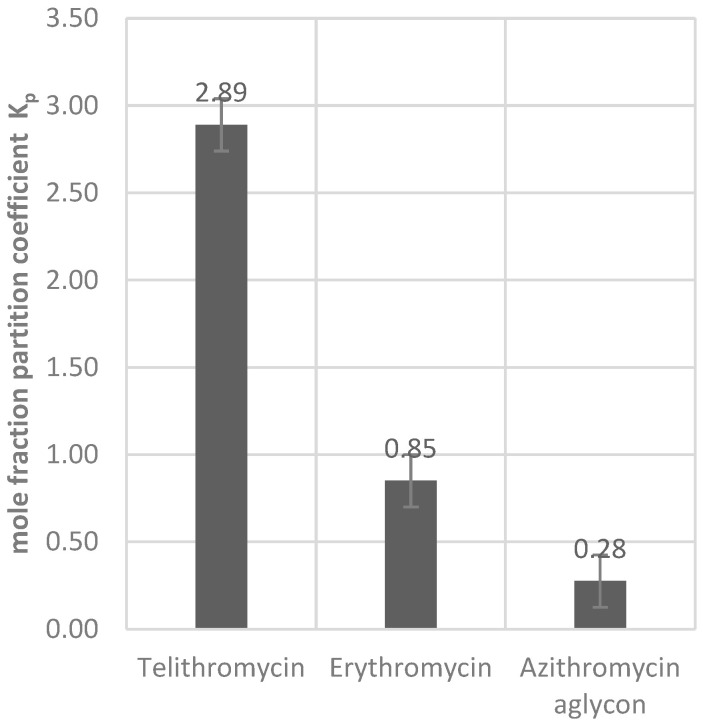
Mole fraction partition coefficients of the selected macrolides and HSA indicate the amount of ligand ound to the protein and thereby the strength of the interaction.

We found that telithromycin shows the strongest binding interaction with HSA among the studied macrolides, exhibiting a mole fraction partition coefficient of 2.89. This is followed by erythromycin (K_p_ = 0.85) and lastly the azithromycin aglycon (K_p_ = 0.22), corresponding to significantly weaker interactions (Figure 3). HSA, as a binding partner, offers several low-affinity binding sites, the majority of which favor hydrophobic interactions. The relatively strong binding of telithromycin to HSA results in significant line broadening. Azithromycin aglycon with 6 acidic protons and no side chains shows the strongest polarity of the compounds studied and exhibits the weakest binding to the protein. Comparing telithromycin and erythromycin in terms of polarity reveals that the fewer acidic protons caused by the introduction of the ketone and the carbamate-attached aromatic side chain result in significantly reduced overall polarity of telithromycin.

Our findings suggest that the interaction between macrolide antibiotics and HSA is largely dependent on the side chain polarity and the specific conformations adopted. The aromatic side chain of telithromycin offers a significant increase in overall conformational flexibility in order to expose or shield moieties to modify parameters such as cell permeability and binding affinities. Additionally, for telithromycin, shielding of the polar moieties is possible by stacking the aromatic side chain with the macrocycle core, making the molecule more prone to apolar interactions. To gain further insight into this phenomenon, we next aimed to elucidate the conformation of telithromycin as the most flexible macrolide antibiotic in the bound state.

### Telithromycin Adopts a Closed Conformation in the Presence of HSA

In order to investigate the conformation of telithromycin bound to HSA to better understand the chameleonic behavior of the drug and to find similarities to previously published bound conformations and solution structures.

To ensure the validity of the acquired transferred NOE data for the ligand in the bound conformation, several conditions must be maintained; NOEs originating from cross relaxation of the free ligand are ideally avoided by choosing a ligand that has a correlation time in the region that results in very small NOEs. Telithromycin, with a molecular mass of 812.02 g/mol, is close enough to the correlation times for which very low NOE signal intensities are expected in D_2_O. Another factor is spin diffusion, which hampers the evaluation of NOE intensities with regard to distances. To avoid issues arising from spin diffusion, short mixing times should be used. For the present investigation, NOE spectra were recorded with a mixing time of 100 ms, which limits spin diffusion significantly.

**Figure 4:**
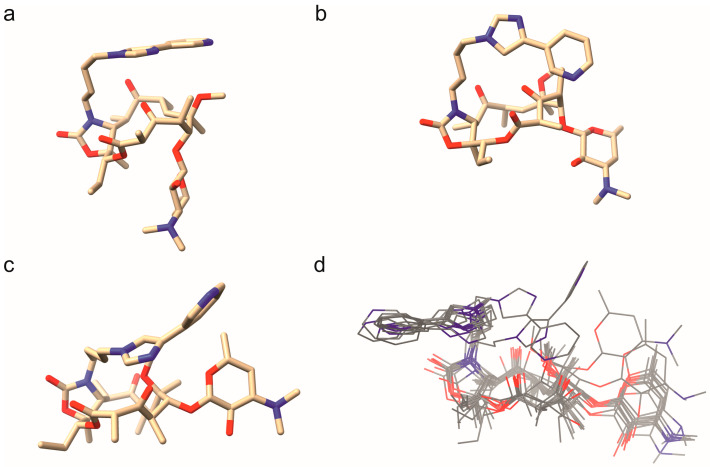
Several conformations of telithromycin. (**a**) First conformation determined via trNOE in the presence of HSA, (**b**) second conformation determined via trNOE with slanted side chain angle, (**c**) conformation taken from pdb entry 4WF9 bound to the 50S ribosomal subunit of *S. aureus*. (**d**) Superposition of published pdb entries of telithromycin bound to ribosomes aligned via the macrocyclic ring [38]. Oxygen atoms are colored red and nitrogen atoms blue.

The observed trNOE crosspeaks indicate the presence of several irreconcilable conformations in the bound state. Here, one conformation is indicated to have its aromatic side chain extended far over the aglycon, showing spatial proximity to the methyl group on the desosamine (Figure 4a). Another main conformation displays the desosamine oriented perpendicularly to the aglycon and the aromatic side chain stacked with the aglycon at a slight angle (Figure 4b). Fewer and less intense crosspeaks (between ethyl and pyridyl) indicate conformations that sport the aromatic side chain oriented away from the aglycon. A full list of the observed trNOE, which are the basis for the structures shown in Figure 4a,b, is given in Table S2. These structures are derived from trNOE distance restraints, which were considered in preliminary force field calculations and subsequently refined in DFT calculations (see Section 3).

The specific carbamate sidechain conformation depicted in Figure 4a is remarkably similar to the crystal structure in pdb entry 4WF9, which shows the ketolide bound to the 50S ribosomal subunit of *S. aureus* (Figure 4c) [39]. The ribosome-bound structure shows a rotated glycosidic bond, resulting in an even tighter conformation, which in our case could not be observed via corresponding trNOEs. Upon close inspection, the aglycon of the deposited structure in Figure 4c shows an inverted stereocenter at the methyl group contained in the β-keto lactone. This indicates the possibility for increased flexibility in the aglycone, as previous studies have suggested, rather low conformational freedom of the macrolactone ring with increasing substitution, as with ketolides [40]. To further characterize the ring conformation, there are two lactone ring conformations described as the 3-endo-folded-out and the corresponding folded-in conformation, where the former is known to occur predominantly in 23S-ribosome-bound systems [41]. We observed the characteristic H4–H11 NOE crosspeak and therefore conclude that the system adopts a 3-endo-folded-out conformation.

Furthermore, our data show the existence of a second conformation in which the aromatic side chain is in close proximity to the ethyl and methoxy group, resulting in an angled pyridyl orientation (Figure 4b). As of now, there is no published PDB structure that matches the second range of observed trNOEs depicted in Figure 4b. The currently known PDB structures mostly display an open conformation with the sidechain extended away from the aglycon (a superposition of all published structures aligned about their aglycon is shown in Figure 4d). Minor conformations represent a transition between the solution structure and the bound conformation and feature the aryl side chain oriented perpendicularly to the aglycon.

To assess the amount of reorganization needed upon TM binding to HSA, i.e., the extent of the structural change between TM in its free and in its two determined HSA-bound states, we conducted a thorough computational conformational analysis on isolated TM (see Section 3 and the Supplementary Materials). Intriguingly, the obtained lowest energy conformers are structurally very similar to the HSA-bound conformation depicted in Figure 4a, judging from the low root mean square deviation (RMSD) below 0.1 Å (Tables S4 and S5) and from visual inspection (Figures S7–S14). This hints that free TM already exists in a geometric arrangement that is ideally disposed to be accepted by an HSA binding site.

**Figure 5:**
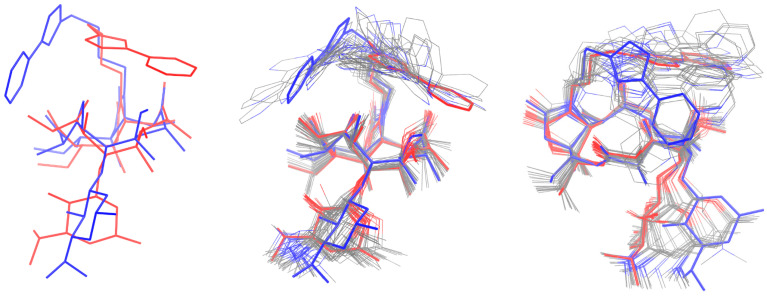
**Left**: Overlay of the structures of the two HSA-bound TM conformers depicted in Figure 4a (red) and Figure 4b (blue); **Middle** and **Right**: Overlay augmented by the first 40 conformers of free TM (ranked according to the B3LYP-D3BJ/def2-SVP@PBE-D3BJ/def2-SVP level of theory, see Section 3) from different angles of view in gray.

Notably, the number of free TM conformers resembling the bound conformation of Figure 4a is much larger than the number of such conformers bearing similarity to the second conformation of HSA-bound TM depicted in Figure 4b (Figures S3–S6). While, as discussed, the former structure also appears to represent the global energetic minimum of free TM, the rarer structural matches to the latter only occur at somewhat higher relative energies (Tables S4 and S5, Figures S6, S27 and S30–S32). Hence, this second conformation is energetically disfavored in the free state and is; thus, less readily available to undergo direct binding to HSA without some preceding geometrical rearrangement of, primarily, the carbamate side chain, albeit the changes required appear overall not too demanding (Figure 5).

Importantly, the two determined conformations of TM in its HSA-bound state both feature the carbamate side chain arranged in a way that shields the polar moieties attached to the aglycon and, thus, enables more hydrophobic interactions, thereby influencing binding to HSA. Notably, the low-energy conformers of free TM all exhibit this side-chain oriented in a similar fashion (Figure 5, Figures S15–S33), all showing a similar shielding arrangement, thereby further backing up the hypothesis that the geometry of free TM is, indeed, ideally predisposed toward HSA binding.

Our findings suggest that telithromycin bound to HSA adopts a variety of conformations, the major ones having the aryl-sidechain superimposed with the aglycon to shield the polar moieties directly attached to the macrocycle. The torsion angle of the glycosidic bond varies greatly between 40 and 140° determined conformations are closely related to conformations known from the literature from co-crystallizations with bacterial ribosomes. There are clear indications for a hydrophobic binding environment, as expected for HSA.

## 3. Materials and Methods

### 3.1. NMR Methods

The Bruker pulse-sequence ledbpgp2s was used for the DOSY measurements. The optimal parameters with regard to signal to noise ratio and reduction in gradient coil strain were determined using a 1D variant of the ledbpgp2s pulse sequence and were as follows: Diffusion time (Δ = 500 ms), diffusion gradient length (δ = 1 ms), gradient strength (g = 80%), number of gradient steps (steps = 32). A linear gradient profile was employed; 1 mM solutions of erythromycin and azithromycin aglycon in D_2_O were prepared, and a DOSY spectrum was recorded. Thereafter, 0.025 mM of HSA was added to the solution, and another spectrum using identical parameters was recorded. The concentration of the telithromycin solution was adjusted according to the solubility of the compound to 0.5 mM telithromycin and 0.0125 mM HSA.

Diffusion coefficients and concentrations of bound and free ligand are related via

(1) $$D_{e}=D_{f}\frac{{[A}_{f}]}{{[A}_{tot}]}+D_{AB}\frac{\left[A_{b}\right]}{{[A}_{tot}]}$$

(2) $${[A}_{tot}]={[A}_{f}]+\left[A_{b}\right]$$

Following up to the previous formula, the mole fraction partition coefficients K_p_ were calculated according to [42]

(3) $$K_{p}=\frac{\left[A_{b}\right]}{{[A}_{f}]}=\frac{D_{e}-D_{f}}{D_{AB}-D_{e}}$$

where A_b_ is the concentration of bound molecules, A_f_ is the concentration of free molecules, and A_tot_ is the total concentration of molecules. K_p_ is indicative of how much of the macrolide is bound to the receptor. D_e_ is the diffusion coefficient of the macrolide in the presence of the protein. D_f_ is the diffusion coefficient of the freely diffusing macrolide. D_AB_ is the diffusion coefficient of the ligand bound to the receptor, which is the diffusion coefficient of the receptor itself.

The trNOE spectra were recorded in a saturated solution of telithromycin and HSA in D_2_O using a mixing time of 100 ms.

All macrolides were obtained from Selvita (Selvita SA, Hexagon, ul. Podole 79, 30-394 Krakow, Poland) and used without further purification. Human serum albumin was purchased from Sigma Aldrich (Sigma Aldrich Handels Gmbh, Marchettigasse 7/2, 1060 Wien, Austria) at the highest available grade. Deuterated solvents were obtained from Eurisotop (Parc des Algorithmes, Bâtiment Homère, 91190 Saint-Aubin, France) and used as received.

The spectra were recorded on a Bruker Avance III 700 MHz spectrometer (Bruker Corporation, 40 Manning Road, Billerica, MA 01821, USA) equipped with a cryogenically cooled TCI probe with z-axis gradients at 298 K. The data were processed in Topspin 4.1.3, and the diffusion coefficients were calculated using exponential fitting in Bruker Dynamics Center 2.8.1.

### 3.2. Computational Methods

For the structures corresponding to the two experimentally observed conformations of HSA-bound telithromycin (Figure 4a,b), initial geometries are obtained by performing a force-field pre-optimization (MM+) [43] taking into account the NOE-derived distance restraints (See Table S2, Figures S1 and S2, distances were set to 5 Å). These geometries were then used as the starting structures for subsequent geometry optimizations (removing the constraints) at the PBE-D3BJ/def2-SVP level of theory employing the COSMO solvation model for water (ε_r_ = 80.1) [44,45,46,47,48,49].

Conformational searches of the telithromycin structure (see Supplementary Materials) were performed with the COSMOconf program in the gas phase [50]. Here, a hierarchy of density functional methods of increasing accuracy was employed to investigate the conformational space to the best possible extent. After the initial generation of a total of 431 conformers employing BALLOON [51], single-point energies were calculated at the PBE-D3BJ/def2-SV(P) level of theory, followed by geometry optimizations at the PBE-D3BJ/def2-SVP level of theory, thereby step by step reducing the overall amount of conformers to 287 structures [44,45,46,51,52]. Subsequently, to more accurately assess the energy of these conformers, B3LYP-D3BJ/def2-SVP single-point calculations and B3LYP-D3BJ/def2-SVP optimizations were carried out (Table S3) [44,45,46,47,48,49]. RMSD analysis of the geometries was carried out with VMD [38].

Additionally, to investigate the effect of the chosen density functional approximation (DFA) and basis set size on the obtained geometry, a selected conformer resembling the structure displayed in Figure 4b (see Supplementary Materials) was reoptimized at the PBE-D3BJ/def2-TZVPPD, B3LYP-D3BJ/def2-SVP, and B3LYP-D3BJ/def2-TZVPPD levels of theory in the gas phase [44,45,46,47,48,49,52]. Moreover, to investigate the effect of the environment on the structure, the same conformers were also reoptimized at the PBE-D3BJ/def2-SVP and PBE-D3BJ/def2-TZVPPD levels of theory while employing the COSMO solvation model for different values of the relative permittivity (ε_r_ = 1.84, 2.38, 8.93, 24.55, 80.1–corresponding to pentane, toluene, DCM, EtOH, and water, Figures S34–S41) [53]. Gratifyingly, all investigated parameters (basis set, DFA, and relative permittivity) exhibit only a very small influence on the resulting optimized geometry

All DFT calculations were performed with TURBOMOLE 7.4.1 [54,55,56]. For speeding up the calculations, the resolution of identity approximation was utilized (RI/RIJK, for PBE and B3LYP calculations, respectively) [57,58,59,60]. Rendered images of the molecular structures were generated with VMD [38].

## 4. Conclusions

The investigated macrolide antibiotics interact with HSA with greatly varying binding strengths, with telithromycin binding the strongest, followed by erythromycin and lastly azithromycin aglycon. The mole fraction partition coefficients give insight into the complex binding interactions and can be adequately determined via DOSY at high precision. Telithromycin adopts a specific conformation upon binding, which shields the polar moieties attached to the aglycon and enables more hydrophobic interactions. Our computational studies show that the solution structures largely match the predominant binding conformations determined by trNOE, indicating that little structural rearrangement is needed between the free and bound states in an aqueous environment. Thus, TM is predisposed towards binding to HSA in the observed conformations.

We here present the first NMR-based study of the interaction strength of a ketolide-antibiotic (telithromycin) and HSA, as well as providing insights into the macrolide conformation in the bound state. The determined conformations represent the first NMR-derived data as well as the first solution-state interaction study of the interaction of macrolides and HSA.

The strong binding interaction between telithromycin and HSA is a potentially relevant factor in the bioavailability and the excretion of the compound. Further investigations are needed to fully understand the underlying mechanisms of the molecular physiology of telithromycin. Therefore, insight into the adverse effects of telithromycin is essential for further developments in this field of research.

## Data Availability

The data presented in this study are available on request from the corresponding author.

## Supplementary Materials

The following supporting information can be downloaded at: https://www.mdpi.com/article/10.3390/ijms262412005/s1.

## Abbreviations

The following abbreviations are used in this manuscript:

HSA	Human serum albumin
TM	Telithromycin
DOSY	Diffusion ordered spectroscopy
NMR	Nuclear magnetic resonance
FA	Fatty acid
PDB	Protein data bank
DFT	Density functional theory
RMSD	Root mean square deviation
DFA	Density functional approximation
(tr)NOE	(transferred) nuclear Overhauser effect
