# Supplementary material for: Investigation of the Binding of the Macrolide Antibiotic Telithromycin to Human Serum Albumin by NMR Spectroscopy
Source: Int J Mol Sci. 2025 Dec 13;26(24):12005. doi: 10.3390/ijms262412005 (PMC12733357; doi:10.3390/ijms262412005)
Supplement: Supplementary file 1 [file ijms-26-12005-s001.zip › ijms-4001646-supplementary.pdf]

Supporting information

Investigation of the binding of the macrolide antibiotic telithromycin to human serum albumin by NMR spectroscopy

Markus Rotzinger [1], Peter Hartmann [1], Barbara Muhry [1], Karina Stadler [1], A. Daniel Boese [1], Predrag Novak[2] and Klaus Zangger [1,*]

[1] University of Graz, Institute of Chemistry, Heinrichstraße 28, Graz, Austria
[2] University of Zagreb, Department of Chemistry, Horvatovac 102a, Zagreb, Croatia
* Correspondence klaus.zangger@uni-graz.at

Table S1 Results of DOSY measurements to study the interaction of macrolide antibiotics with HSA

**Macrolide**	**D_f [E-10 m²/s]**	**D_e [E-10 m²/s]**	**K_p**
Telithromycin	4.13 ± 0.06	1.56 ± 0.17	2.89
Erythromycin	4.30 ± 0.11	2.63 ± 0.1	0.85
Azithromycin aglycon	5.67 ± 0.1	4.59 ± 0.12	0.28
Human serum albumin	0.67 ± 0.03		

D_f…..diffusion coefficient of the free macrolide

D_e…..diffusion coefficient of the macrolide in the presence of HAS

K_p…..mole fraction partition coefficient

Convection suppression:

Convection can be a major factor for systematic errors in diffusion experiments in the slow motion regime, which can be alleviated in several ways.[57] Usually, convection is compensated for in diffusion measurements by including a third spoil gradient (ledbpgp3s). A trial run employing this variety of diffusion experiments showed that, for our application, convection was responsible for only a negligible change in absolute diffusion coefficients, while the sensitivity of the experiment is significantly reduced in comparison with the two-spoil gradient variant (ledbpgp2s). The only small impact of convection in our study can be explained by the innate physical traits of D_2O which reduce convection effects significantly in comparison with $CDCl_3$. Due to the limited solubility of macrolide antibiotics and the resulting low sensitivity, all diffusion measurements were carried out employing the ledbpgp2s sequence.

Table S2. Telithromycin ^{1}H assignment and transferred NOE crosspeaks in the presence of HSA.

**Macrocycle**	**δ ^{1}H**	**NOE crosspeaks**	**sidechain**	**δ ^{1}H**	**NOE crosspeaks**
1			16		
2	4.17		17	3.6	10-Me, 18, 19
2-Me	1.23	6-Me, 6-OMe, 10 ,11 ,13 ,14	18	1.47	6-OMe, 17, 19, 20, 23
3			19	1.78; 1.85	17, 18, 19, 21, 25
4	3.15	4-Me, 5, 6-OMe, 7, 11	20	4.06; 4.16	18, 21, 23, 25,
4-Me	1.2	4, 8, 10, 11, 12-Me, 21, 23, 25, 28, 29	21	7.65	4-Me, 6-Me, 6-OMe, 14, 15, 17, 18, 19, 20
5	3.99	4, 4-Me, 6-Me, 6-OMe, 29	22		
6			23	7.79	4-Me, 6-Me, 6-OMe, 10-Me, 14, 15, 17, 18, 19, 20
6-Me	1.13	2-Me,5, 6-OMe, 8	24		
6-OMe	2.09	5, 6-Me, 12-Me,18, 21, 23, 25, 26, 28, 30, 31	25	8.82	4-Me, 6-Me, 6-OMe, 10, 10-Me, 11, 14, 15, 17, 18, 19, 20, 31-N(Me)$_2$
7	1.67; 1.75	4, 8	26	8.44	4-Me, 10-Me, 15, 33Me
8	2.53	4-Me, 6-Me, 7, 10-Me	27	7.51	25, 26, 28
8-Me	1.18	7, 8, 10, 10-Me	28	8.16	4-Me, 10-Me, 28, 33-Me
9			29	4.34	4-Me, 5, 11, 33
10	3.23	4-Me, 7, 8, 10-Me, 12-Me, 13	30	3.43	6-OMe, 29, 31-N(Me)2, 32, 33
10-Me	0.99	2-Me, 8, 8-Me, 10, 11, 12-Me, 17, 25, 26, 28	31	2.87	6-Me, 6-OMe, 12-Me, 29, 30, 33
11	3.48	4-Me, 6-OMe, 10-Me, 12-Me, 13, 18	31-N(Me)$_2$	2.78	4-Me, 6-OMe, 29, 30, 32
12			32	1.51; 2.10	29, 31, 31-N(Me)2
12-Me	1.58	4-Me, 6-OMe, 10, 10-Me, 11, 14, 15	33	3.78	6-OMe, 12-Me, 29, 30, 31, 33-Me
13	4.7	10, 11	33-Me	1.32	6-OMe, 8-Me, 12-Me
14	1.63; 1.82	2-Me, 12-Me, 21, 25			
15	0.8	2-Me, 19, 21, 23, 25, 28			

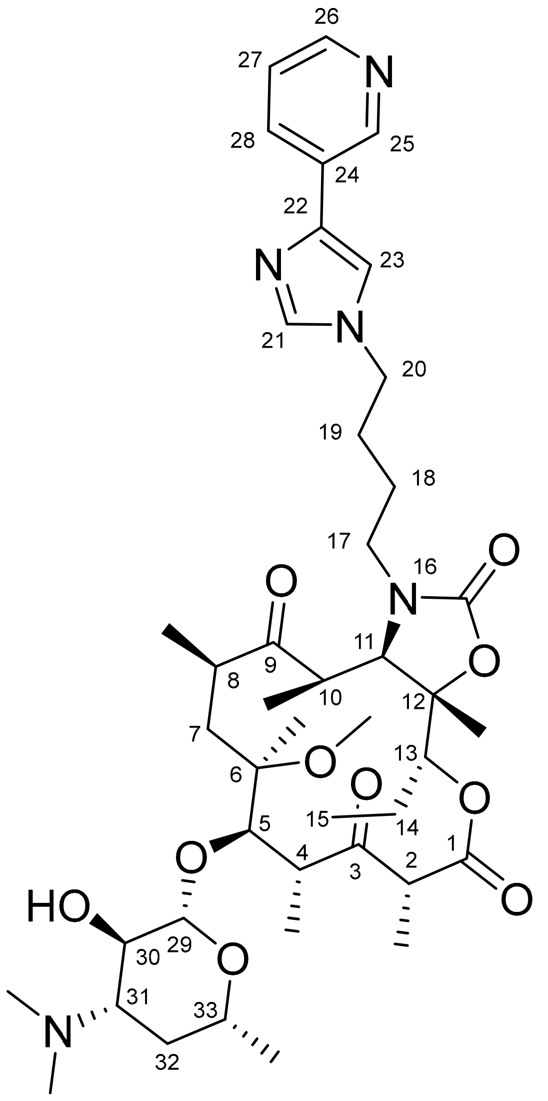

Figure S1: Telithromycin

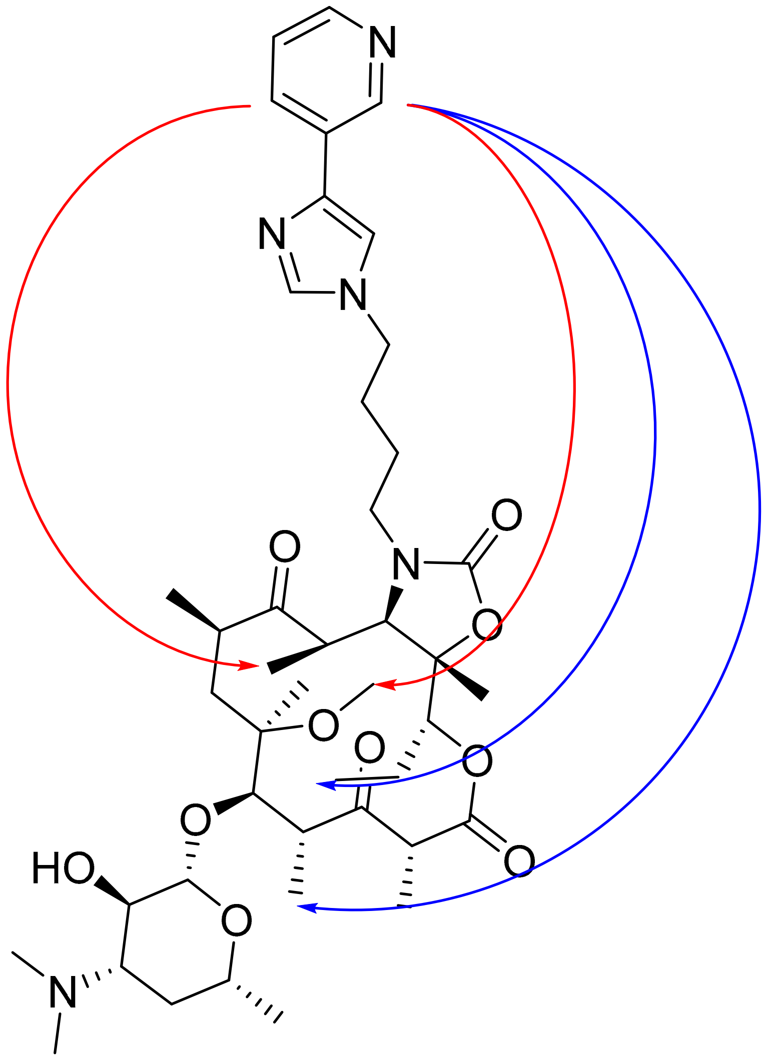

Figure S2. Telithromycin with arrows indicating the present trNOEs. Blue arrows correspond to a conformation where the sidechain is folded over the back of the aglycone while red arrows correspond to a conformation where the sidechain is in closer proximity to the front of the aglycone.

Conformational Analysis Data

Table S3. Calculated conformers ordered after their relative electronic energies as obtained from the conformational analysis, carried out as described in the "Methods" section of the main text. Left: ordered after the PBE-D3BJ/def2-SVP optimized electronic energies; right: ordered after the B3LYP-D3BJ/def2-SVP@PBE-D3BJ/def2-SVP electronic energies. The lowest energy conformer for each method was taken as the reference in each case.

PBE rank	B3LYP rank	ΔE_{el} PBE-D3BJ/ def2-SVP optimized [kJ/mol]	ΔE_{el} B3LYP-D3BJ/ def2-SVP single point [kJ/mol]
0	0	0.00	0.00
1	2	0.77	1.49
2	1	1.08	0.62
3	3	2.09	2.21
4	10	2.63	6.50
5	5	3.42	4.00
6	4	3.61	3.24
7	14	4.59	8.12
8	7	5.09	5.72
9	19	5.89	11.30
10	13	6.07	8.02
11	8	6.08	5.80
12	11	6.11	6.53
13	6	6.59	4.83
14	12	6.95	7.11
15	9	7.99	6.32
16	15	8.51	8.31
17	34	9.46	13.48
18	17	9.62	10.72
19	20	9.82	11.40
20	21	9.92	11.73
21	32	10.24	13.37
22	25	10.30	12.40
23	18	10.72	11.03
24	30	11.04	12.81

B3LYP rank	PBE rank	ΔE_{el} PBE-D3BJ/ def2-SVP optimized [kJ/mol]	ΔE_{el} B3LYP-D3BJ/ def2-SVP single point [kJ/mol]
0	0	0.00	0.00
1	2	1.08	0.62
2	1	0.77	1.49
3	3	2.09	2.21
4	6	3.61	3.24
5	5	3.42	4.00
6	13	6.59	4.83
7	8	5.09	5.72
8	11	6.08	5.80
9	15	7.99	6.32
10	4	2.63	6.50
11	12	6.11	6.53
12	14	6.95	7.11
13	10	6.07	8.02
14	7	4.59	8.12
15	16	8.51	8.31
16	30	11.44	9.47
17	18	9.62	10.72
18	23	10.72	11.03
19	9	5.89	11.30
20	19	9.82	11.40
21	20	9.92	11.73
22	41	12.55	11.77
23	52	14.08	11.85
24	31	11.49	12.30

25	28	11.16	12.76
26	36	11.23	13.89
27	37	11.35	14.14
28	41	11.36	14.96
29	38	11.41	14.27
30	16	11.44	9.47
31	24	11.49	12.30
32	49	11.64	15.68
33	31	11.84	12.96
34	47	11.86	15.52
35	40	11.88	14.56
36	54	11.89	16.45
37	50	11.89	15.81
38	60	11.98	17.23
39	42	12.02	15.09
40	27	12.54	12.60
41	22	12.55	11.77
42	51	12.61	16.16
43	52	12.71	16.36
44	59	12.98	16.92
45	39	13.18	14.43
46	65	13.27	17.93
47	43	13.37	15.21
48	26	13.63	12.48
49	45	13.64	15.35
50	79	13.64	19.49
51	29	13.73	12.77
52	23	14.08	11.85
53	61	14.15	17.39
54	62	14.31	17.64
55	56	14.71	16.61
56	69	14.71	18.74
57	33	14.72	13.39
58	35	14.81	13.55
59	55	14.97	16.59

25	22	10.30	12.40
26	48	13.63	12.48
27	40	12.54	12.60
28	25	11.16	12.76
29	51	13.73	12.77
30	24	11.04	12.81
31	33	11.84	12.96
32	21	10.24	13.37
33	57	14.72	13.39
34	17	9.46	13.48
35	58	14.81	13.55
36	26	11.23	13.89
37	27	11.35	14.14
38	29	11.41	14.27
39	45	13.18	14.43
40	35	11.88	14.56
41	28	11.36	14.96
42	39	12.02	15.09
43	47	13.37	15.21
44	68	16.04	15.25
45	49	13.64	15.35
46	72	16.27	15.51
47	34	11.86	15.52
48	62	15.42	15.54
49	32	11.64	15.68
50	37	11.89	15.81
51	42	12.61	16.16
52	43	12.71	16.36
53	60	15.20	16.41
54	36	11.89	16.45
55	59	14.97	16.59
56	55	14.71	16.61
57	61	15.35	16.82
58	64	15.74	16.89
59	44	12.98	16.92

60	53	15.20	16.41
61	57	15.35	16.82
62	48	15.42	15.54
63	72	15.48	19.07
64	58	15.74	16.89
65	66	15.84	18.29
66	71	15.84	19.02
67	81	15.97	19.63
68	44	16.04	15.25
69	83	16.05	19.92
70	73	16.08	19.16
71	86	16.17	20.17
72	46	16.27	15.51
73	74	16.49	19.29
74	78	16.58	19.49
75	87	16.72	20.21
76	70	16.98	18.95
77	85	17.01	20.17
78	101	17.11	22.97
79	91	17.18	21.30
80	77	17.19	19.48
81	75	17.31	19.45
82	76	17.32	19.48
83	94	17.37	21.55
84	82	17.37	19.76
85	88	17.42	20.67
86	92	17.82	21.39
87	68	17.85	18.69
88	97	17.95	22.10
89	108	18.07	23.40
90	64	18.20	17.92
91	63	18.21	17.83
92	96	18.21	22.01
93	89	18.50	20.84
94	119	18.63	24.63

60	38	11.98	17.23
61	53	14.15	17.39
62	54	14.31	17.64
63	91	18.21	17.83
64	90	18.20	17.92
65	46	13.27	17.93
66	65	15.84	18.29
67	95	18.80	18.35
68	87	17.85	18.69
69	56	14.71	18.74
70	76	16.98	18.95
71	66	15.84	19.02
72	63	15.48	19.07
73	70	16.08	19.16
74	73	16.49	19.29
75	81	17.31	19.45
76	82	17.32	19.48
77	80	17.19	19.48
78	74	16.58	19.49
79	50	13.64	19.49
80	104	19.93	19.49
81	67	15.97	19.63
82	84	17.37	19.76
83	69	16.05	19.92
84	99	19.07	20.05
85	77	17.01	20.17
86	71	16.17	20.17
87	75	16.72	20.21
88	85	17.42	20.67
89	93	18.50	20.84
90	101	19.75	21.26
91	79	17.18	21.30
92	86	17.82	21.39
93	109	20.23	21.42
94	83	17.37	21.55

95	67	18.80	18.35
96	109	18.81	23.43
97	95	18.89	21.96
98	121	18.97	24.92
99	84	19.07	20.05
100	123	19.35	25.09
101	90	19.75	21.26
102	104	19.77	23.21
103	100	19.90	22.87
104	80	19.93	19.49
105	99	20.00	22.65
106	125	20.02	25.41
107	118	20.17	24.54
108	120	20.20	24.86
109	93	20.23	21.42
110	126	20.66	25.49
111	98	20.73	22.22
112	124	20.82	25.20
113	139	21.00	27.41
114	102	21.00	23.04
115	112	21.03	23.67
116	114	21.04	24.05
117	103	21.14	23.09
118	111	21.34	23.62
119	115	21.39	24.08
120	128	21.42	25.77
121	105	21.62	23.21
122	117	21.64	24.42
123	142	21.67	27.75
124	116	21.77	24.39
125	113	21.78	23.77
126	133	21.97	26.73
127	107	22.11	23.37
128	122	22.31	24.99
129	110	22.36	23.60

95	97	18.89	21.96
96	92	18.21	22.01
97	88	17.95	22.10
98	111	20.73	22.22
99	105	20.00	22.65
100	103	19.90	22.87
101	78	17.11	22.97
102	114	21.00	23.04
103	117	21.14	23.09
104	102	19.77	23.21
105	121	21.62	23.21
106	143	24.60	23.23
107	127	22.11	23.37
108	89	18.07	23.40
109	96	18.81	23.43
110	129	22.36	23.60
111	118	21.34	23.62
112	115	21.03	23.67
113	125	21.78	23.77
114	116	21.04	24.05
115	119	21.39	24.08
116	124	21.77	24.39
117	122	21.64	24.42
118	107	20.17	24.54
119	94	18.63	24.63
120	108	20.20	24.86
121	98	18.97	24.92
122	128	22.31	24.99
123	100	19.35	25.09
124	112	20.82	25.20
125	106	20.02	25.41
126	110	20.66	25.49
127	133	23.10	25.52
128	120	21.42	25.77
129	135	23.32	26.04

130	147	22.62	28.74
131	132	22.83	26.63
132	137	23.04	26.95
133	127	23.10	25.52
134	149	23.20	29.21
135	129	23.32	26.04
136	136	23.50	26.92
137	138	23.65	27.26
138	134	23.72	26.83
139	143	23.73	27.91
140	135	23.88	26.85
141	130	24.06	26.19
142	131	24.40	26.19
143	106	24.60	23.23
144	161	24.67	30.73
145	146	24.70	28.73
146	140	24.86	27.46
147	150	25.18	29.42
148	148	25.53	29.10
149	153	25.73	29.79
150	162	26.05	30.93
151	155	26.35	29.99
152	158	26.66	30.53
153	145	26.75	28.31
154	144	26.84	28.19
155	154	27.04	29.95
156	160	27.07	30.72
157	141	27.51	27.57
158	156	27.53	30.35
159	173	27.96	32.91
160	177	28.18	33.22
161	151	28.23	29.62
162	157	28.36	30.46
163	152	28.38	29.68
164	189	28.53	34.75

130	141	24.06	26.19
131	142	24.40	26.19
132	131	22.83	26.63
133	126	21.97	26.73
134	138	23.72	26.83
135	140	23.88	26.85
136	136	23.50	26.92
137	132	23.04	26.95
138	137	23.65	27.26
139	113	21.00	27.41
140	146	24.86	27.46
141	157	27.51	27.57
142	123	21.67	27.75
143	139	23.73	27.91
144	154	26.84	28.19
145	153	26.75	28.31
146	145	24.70	28.73
147	130	22.62	28.74
148	148	25.53	29.10
149	134	23.20	29.21
150	147	25.18	29.42
151	161	28.23	29.62
152	163	28.38	29.68
153	149	25.73	29.79
154	155	27.04	29.95
155	151	26.35	29.99
156	158	27.53	30.35
157	162	28.36	30.46
158	152	26.66	30.53
159	189	31.17	30.58
160	156	27.07	30.72
161	144	24.67	30.73
162	150	26.05	30.93
163	183	30.13	31.17
164	185	30.34	31.50

165	175	28.55	33.06
166	170	28.60	32.48
167	179	28.91	33.86
168	168	29.05	32.25
169	166	29.11	31.82
170	184	29.18	34.36
171	186	29.25	34.62
172	181	29.59	33.89
173	172	29.63	32.83
174	182	29.67	34.04
175	169	29.74	32.33
176	165	29.81	31.54
177	185	29.88	34.37
178	191	29.89	34.83
179	171	29.99	32.65
180	196	29.99	36.49
181	174	30.03	32.97
182	197	30.09	36.53
183	163	30.13	31.17
184	176	30.23	33.11
185	164	30.34	31.50
186	180	30.37	33.87
187	178	30.59	33.70
188	194	30.80	36.05
189	159	31.17	30.58
190	198	31.40	36.74
191	167	31.42	32.25
192	187	31.47	34.72
193	183	31.78	34.35
194	188	32.00	34.73
195	190	32.23	34.78
196	193	33.02	35.44
197	204	33.31	38.76
198	199	33.46	36.87
199	195	33.52	36.36

165	176	29.81	31.54
166	169	29.11	31.82
167	191	31.42	32.25
168	168	29.05	32.25
169	175	29.74	32.33
170	166	28.60	32.48
171	179	29.99	32.65
172	173	29.63	32.83
173	159	27.96	32.91
174	181	30.03	32.97
175	165	28.55	33.06
176	184	30.23	33.11
177	160	28.18	33.22
178	187	30.59	33.70
179	167	28.91	33.86
180	186	30.37	33.87
181	172	29.59	33.89
182	174	29.67	34.04
183	193	31.78	34.35
184	170	29.18	34.36
185	177	29.88	34.37
186	171	29.25	34.62
187	192	31.47	34.72
188	194	32.00	34.73
189	164	28.53	34.75
190	195	32.23	34.78
191	178	29.89	34.83
192	212	36.48	35.40
193	196	33.02	35.44
194	188	30.80	36.05
195	199	33.52	36.36
196	180	29.99	36.49
197	182	30.09	36.53
198	190	31.40	36.74
199	198	33.46	36.87

200	207	33.76	39.31
201	203	34.43	38.18
202	202	35.04	37.95
203	215	35.10	41.32
204	205	35.11	38.90
205	217	35.18	42.02
206	209	35.64	39.71
207	206	35.68	38.97
208	200	35.83	37.21
209	201	35.92	37.77
210	210	36.18	40.23
211	208	36.27	39.41
212	192	36.48	35.40
213	218	37.09	42.06
214	214	37.44	41.23
215	220	37.69	42.35
216	228	38.02	44.22
217	229	38.05	44.37
218	230	38.19	44.73
219	211	38.33	40.29
220	213	38.41	40.86
221	226	38.59	43.65
222	232	39.12	45.39
223	222	39.15	42.65
224	212	39.30	40.39
225	231	39.53	45.28
226	227	39.53	43.73
227	223	39.85	43.10
228	233	40.66	45.97
229	238	40.67	47.13
230	216	40.68	41.81
231	224	40.89	43.18
232	241	41.26	47.85
233	239	41.69	47.22
234	240	41.85	47.71

200	208	35.83	37.21
201	209	35.92	37.77
202	202	35.04	37.95
203	201	34.43	38.18
204	197	33.31	38.76
205	204	35.11	38.90
206	207	35.68	38.97
207	200	33.76	39.31
208	211	36.27	39.41
209	206	35.64	39.71
210	210	36.18	40.23
211	219	38.33	40.29
212	224	39.30	40.39
213	220	38.41	40.86
214	214	37.44	41.23
215	203	35.10	41.32
216	230	40.68	41.81
217	205	35.18	42.02
218	213	37.09	42.06
219	241	45.02	42.19
220	215	37.69	42.35
221	237	43.47	42.35
222	223	39.15	42.65
223	227	39.85	43.10
224	231	40.89	43.18
225	242	45.15	43.20
226	221	38.59	43.65
227	226	39.53	43.73
228	216	38.02	44.22
229	217	38.05	44.37
230	218	38.19	44.73
231	225	39.53	45.28
232	222	39.12	45.39
233	228	40.66	45.97
234	248	46.36	46.18

235	237	42.12	47.08
236	242	43.15	48.10
237	221	43.47	42.35
238	236	43.69	46.58
239	243	44.05	48.21
240	249	44.30	50.80
241	219	45.02	42.19
242	225	45.15	43.20
243	247	45.60	50.20
244	246	45.80	50.19
245	250	46.02	51.94
246	251	46.08	52.02
247	245	46.21	49.87
248	234	46.36	46.18
249	235	47.40	46.43
250	253	47.47	53.92
251	254	47.64	53.96
252	248	48.70	50.68
253	244	48.85	48.22
254	252	49.12	52.90
255	255	51.66	54.16
256	261	51.69	58.13
257	260	52.10	57.55
258	257	52.20	55.17
259	262	52.29	58.73
260	256	53.17	54.62
261	259	53.18	55.80
262	258	53.21	55.77
263	263	58.44	59.95
264	264	60.39	65.08
265	265	61.72	66.46
266	267	63.92	70.07
267	268	65.05	70.53
268	266	70.58	68.50
269	269	71.88	77.85

235	249	47.40	46.43
236	238	43.69	46.58
237	235	42.12	47.08
238	229	40.67	47.13
239	233	41.69	47.22
240	234	41.85	47.71
241	232	41.26	47.85
242	236	43.15	48.10
243	239	44.05	48.21
244	253	48.85	48.22
245	247	46.21	49.87
246	244	45.80	50.19
247	243	45.60	50.20
248	252	48.70	50.68
249	240	44.30	50.80
250	245	46.02	51.94
251	246	46.08	52.02
252	254	49.12	52.90
253	250	47.47	53.92
254	251	47.64	53.96
255	255	51.66	54.16
256	260	53.17	54.62
257	258	52.20	55.17
258	262	53.21	55.77
259	261	53.18	55.80
260	257	52.10	57.55
261	256	51.69	58.13
262	259	52.29	58.73
263	263	58.44	59.95
264	264	60.39	65.08
265	265	61.72	66.46
266	268	70.58	68.50
267	266	63.92	70.07
268	267	65.05	70.53
269	269	71.88	77.85

270	270	72.01	80.02
271	271	84.65	89.58
272	273	87.02	91.79
273	272	88.07	91.69
274	275	89.67	95.45
275	274	90.88	94.12
276	276	92.83	95.78
277	277	92.93	97.67
278	278	95.70	98.80
279	279	102.16	108.18
280	280	109.04	112.28
281	281	110.12	115.73
282	282	115.05	116.89
283	283	115.67	118.86
284	285	119.64	128.15
285	284	122.14	124.45
286	286	131.88	138.82

270	270	72.01	80.02
271	271	84.65	89.58
272	273	88.07	91.69
273	272	87.02	91.79
274	275	90.88	94.12
275	274	89.67	95.45
276	276	92.83	95.78
277	277	92.93	97.67
278	278	95.70	98.80
279	279	102.16	108.18
280	280	109.04	112.28
281	281	110.12	115.73
282	282	115.05	116.89
283	283	115.67	118.86
284	285	122.14	124.45
285	284	119.64	128.15
286	286	131.88	138.82

Table S4. RMSDs of the first 90 conformers ordered after their relative electronic energies obtained from the PBE-D3BJ/def2-SVP optimizations during the conformational analysis (see "Methods" section of the main text) vs the structures of the two conformations depicted in Figure 4a and 4b. Conformers exhibiting an RMSD below 0.1 Å are highlighted in bold.

PBE rank	B3LYP rank	ΔE_{el} PBE-D3BJ/ def2-SVP optimized [kJ/mol]	ΔE_{el} B3LYP-D3BJ/ def2-SVP single point [kJ/mol]	RMSD vs the structure depicted in Figure 4a [Å]	RMSD vs structure depicted in Figure 4b [Å]
0	0	0.00	0.00	**0.0986**	0.4337
1	2	0.77	1.49	**0.0979**	0.4919
2	1	1.08	0.62	**0.0370**	0.4436
3	3	2.09	2.21	0.1025	0.4282
4	10	2.63	6.50	0.2281	0.5130
5	5	3.42	4.00	0.2468	0.3483
6	4	3.61	3.24	0.2378	0.4875
7	14	4.59	8.12	**0.0913**	0.3942
8	7	5.09	5.72	0.2262	0.5194
9	19	5.89	11.30	0.3613	0.1684
10	13	6.07	8.02	**0.0849**	0.4699
11	8	6.08	5.80	0.4039	0.1184
12	11	6.11	6.53	0.2211	0.5076
13	6	6.59	4.83	**0.0977**	0.3933
14	12	6.95	7.11	**0.0558**	0.4417
15	9	7.99	6.32	0.3682	0.1724
16	15	8.51	8.31	0.1082	0.3841
17	34	9.46	13.48	0.4362	0.1128
18	17	9.62	10.72	0.4004	0.1273
19	20	9.82	11.40	0.2464	0.3416
20	21	9.92	11.73	0.5276	0.1748
21	32	10.24	13.37	0.6011	0.2394
22	25	10.30	12.40	0.3994	0.1286
23	18	10.72	11.03	**0.0810**	0.4344
24	30	11.04	12.81	0.2318	0.5002
25	28	11.16	12.76	**0.0910**	0.4281
26	36	11.23	13.89	0.1075	0.4252
27	37	11.35	14.14	0.1821	0.2790

28	41	11.36	14.96	0.3602	0.1268
29	38	11.41	14.27	0.1793	0.3709
30	16	11.44	9.47	0.1678	0.4460
31	24	11.49	12.30	**0.0593**	0.4140
32	49	11.64	15.68	0.6074	0.2445
33	31	11.84	12.96	0.1485	0.3881
34	47	11.86	15.52	0.2220	0.2583
35	40	11.88	14.56	**0.0803**	0.4379
36	54	11.89	16.45	**0.0781**	0.3714
37	50	11.89	15.81	0.4055	**0.0889**
38	60	11.98	17.23	**0.0408**	0.4164
39	42	12.02	15.09	**0.0904**	0.4371
40	27	12.54	12.60	0.2217	0.2567
41	22	12.55	11.77	0.3421	0.1622
42	51	12.61	16.16	**0.0909**	0.4583
43	52	12.71	16.36	**0.0810**	0.4238
44	59	12.98	16.92	0.5295	0.1815
45	39	13.18	14.43	0.2352	0.2522
46	65	13.27	17.93	**0.0921**	0.4025
47	43	13.37	15.21	**0.0809**	0.4177
48	26	13.63	12.48	0.4641	0.1477
49	45	13.64	15.35	0.2257	0.5078
50	79	13.64	19.49	**0.0533**	0.4025
51	29	13.73	12.77	0.1254	0.3650
52	23	14.08	11.85	0.1966	0.3798
53	61	14.15	17.39	**0.0960**	0.4281
54	62	14.31	17.64	**0.0935**	0.4321
55	56	14.71	16.61	0.4606	0.2108
56	69	14.71	18.74	0.3846	**0.0983**
57	33	14.72	13.39	0.1239	0.3528
58	35	14.81	13.55	**0.0783**	0.3904
59	55	14.97	16.59	0.4554	0.2916
60	53	15.20	16.41	0.1783	0.3714
61	57	15.35	16.82	0.4803	0.2048
62	48	15.42	15.54	0.2007	0.2796

63	72	15.48	19.07	0.4959	0.1811
64	58	15.74	16.89	0.2239	0.3675
65	66	15.84	18.29	0.5275	0.2277
66	71	15.84	19.02	0.5165	0.1917
67	81	15.97	19.63	0.2548	0.3275
68	44	16.04	15.25	0.4406	0.1056
69	83	16.05	19.92	0.4638	0.1693
70	73	16.08	19.16	0.4080	0.1296
71	86	16.17	20.17	**0.0323**	0.4175
72	46	16.27	15.51	0.1543	0.3712
73	74	16.49	19.29	0.4705	0.1751
74	78	16.58	19.49	**0.0723**	0.4172
75	87	16.72	20.21	**0.0943**	0.4281
76	70	16.98	18.95	0.4094	0.1303
77	85	17.01	20.17	0.4859	0.1423
78	77	17.19	19.48	**0.0438**	0.4103
79	75	17.31	19.45	0.4670	0.1074
80	76	17.32	19.48	**0.0807**	0.4198
81	82	17.37	19.76	0.1078	0.3848
82	88	17.42	20.67	0.4882	0.1585
83	68	17.85	18.69	0.5195	0.2092
84	64	18.20	17.92	0.2690	0.4974
85	63	18.21	17.83	**0.0983**	0.4251
86	89	18.50	20.84	0.2098	0.2648
87	67	18.80	18.35	0.1126	0.3781
88	84	19.07	20.05	0.1214	0.3588
89	80	19.93	19.49	0.5332	0.2231

Table S5. RMSDs of the first 90 conformers ordered after their relative electronic energies obtained from the B3LYP-D3BJ/def2-SVP@PBE-D3BJ/def2-SVP single point calculations during the conformational analysis (see “Methods” section of the main text) vs the structures of the two conformations depicted in Figure 4a and 4b. Conformers exhibiting an RMSD below 0.1 Å are highlighted in bold.

B3LYP rank	PBE rank	ΔE_{el} PBE-D3BJ/ def2-SVP optimized [kJ/mol]	ΔE_{el} B3LYP-D3BJ/ def2-SVP single point [kJ/mol]	RMSD vs the structure depicted in Figure 4a [Å]	RMSD vs structure depicted in Figure 4b [Å]
0	0	0.00	0.00	**0.0986**	0.4337
1	2	1.08	0.62	**0.0370**	0.4436
2	1	0.77	1.49	**0.0979**	0.4919
3	3	2.09	2.21	0.1025	0.4282
4	6	3.61	3.24	0.2378	0.4875
5	5	3.42	4.00	0.2468	0.3483
6	13	6.59	4.83	**0.0977**	0.3933
7	8	5.09	5.72	0.2262	0.5194
8	11	6.08	5.80	0.4039	0.1184
9	15	7.99	6.32	0.3682	0.1724
10	4	2.63	6.50	0.2281	0.5130
11	12	6.11	6.53	0.2211	0.5076
12	14	6.95	7.11	**0.0558**	0.4417
13	10	6.07	8.02	**0.0849**	0.4699
14	7	4.59	8.12	**0.0913**	0.3942
15	16	8.51	8.31	0.1082	0.3841
16	30	11.44	9.47	0.1678	0.4460
17	18	9.62	10.72	0.4004	0.1273
18	23	10.72	11.03	**0.0810**	0.4344
19	9	5.89	11.30	0.3613	0.1684
20	19	9.82	11.40	0.2464	0.3416
21	20	9.92	11.73	0.5276	0.1748
22	41	12.55	11.77	0.3421	0.1622
23	52	14.08	11.85	0.1966	0.3798
24	31	11.49	12.30	**0.0593**	0.4140
25	22	10.30	12.40	0.3994	0.1286
26	48	13.63	12.48	0.4641	0.1477

27	40	12.54	12.60	0.2217	0.2567
28	25	11.16	12.76	**0.0910**	0.4281
29	51	13.73	12.77	0.1254	0.3650
30	24	11.04	12.81	0.2318	0.5002
31	33	11.84	12.96	0.1485	0.3881
32	21	10.24	13.37	0.6011	0.2394
33	57	14.72	13.39	0.1239	0.3528
34	17	9.46	13.48	0.4362	0.1128
35	58	14.81	13.55	**0.0783**	0.3904
36	26	11.23	13.89	0.1075	0.4252
37	27	11.35	14.14	0.1821	0.2790
38	29	11.41	14.27	0.1793	0.3709
39	45	13.18	14.43	0.2352	0.2522
40	35	11.88	14.56	**0.0803**	0.4379
41	28	11.36	14.96	0.3602	0.1268
42	39	12.02	15.09	**0.0904**	0.4371
43	47	13.37	15.21	**0.0809**	0.4177
44	68	16.04	15.25	0.4406	0.1056
45	49	13.64	15.35	0.2257	0.5078
46	72	16.27	15.51	0.1543	0.3712
47	34	11.86	15.52	0.2220	0.2583
48	62	15.42	15.54	0.2007	0.2796
49	32	11.64	15.68	0.6074	0.2445
50	37	11.89	15.81	0.4055	**0.0889**
51	42	12.61	16.16	**0.0909**	0.4583
52	43	12.71	16.36	**0.0810**	0.4238
53	60	15.20	16.41	0.1783	0.3714
54	36	11.89	16.45	**0.0781**	0.3714
55	59	14.97	16.59	0.4554	0.2916
56	55	14.71	16.61	0.4606	0.2108
57	61	15.35	16.82	0.4803	0.2048
58	64	15.74	16.89	0.2239	0.3675
59	44	12.98	16.92	0.5295	0.1815
60	38	11.98	17.23	**0.0408**	0.4164
61	53	14.15	17.39	**0.0960**	0.4281

62	54	14.31	17.64	**0.0935**	0.4321
63	91	18.21	17.83	**0.0983**	0.4251
64	90	18.20	17.92	0.2690	0.4974
65	46	13.27	17.93	**0.0921**	0.4025
66	65	15.84	18.29	0.5275	0.2277
67	95	18.80	18.35	0.1126	0.3781
68	87	17.85	18.69	0.5195	0.2092
69	56	14.71	18.74	0.3846	**0.0983**
70	76	16.98	18.95	0.4094	0.1303
71	66	15.84	19.02	0.5165	0.1917
72	63	15.48	19.07	0.4959	0.1811
73	70	16.08	19.16	0.4080	0.1296
74	73	16.49	19.29	0.4705	0.1751
75	81	17.31	19.45	0.4670	0.1074
76	82	17.32	19.48	**0.0807**	0.4198
77	80	17.19	19.48	**0.0438**	0.4103
78	74	16.58	19.49	**0.0723**	0.4172
79	50	13.64	19.49	**0.0533**	0.4025
80	104	19.93	19.49	0.5332	0.2231
81	67	15.97	19.63	0.2548	0.3275
82	84	17.37	19.76	0.1078	0.3848
83	69	16.05	19.92	0.4638	0.1693
84	99	19.07	20.05	0.1214	0.3588
85	77	17.01	20.17	0.4859	0.1423
86	71	16.17	20.17	**0.0323**	0.4175
87	75	16.72	20.21	**0.0943**	0.4281
88	85	17.42	20.67	0.4882	0.1585
89	93	18.50	20.84	0.2098	0.2648

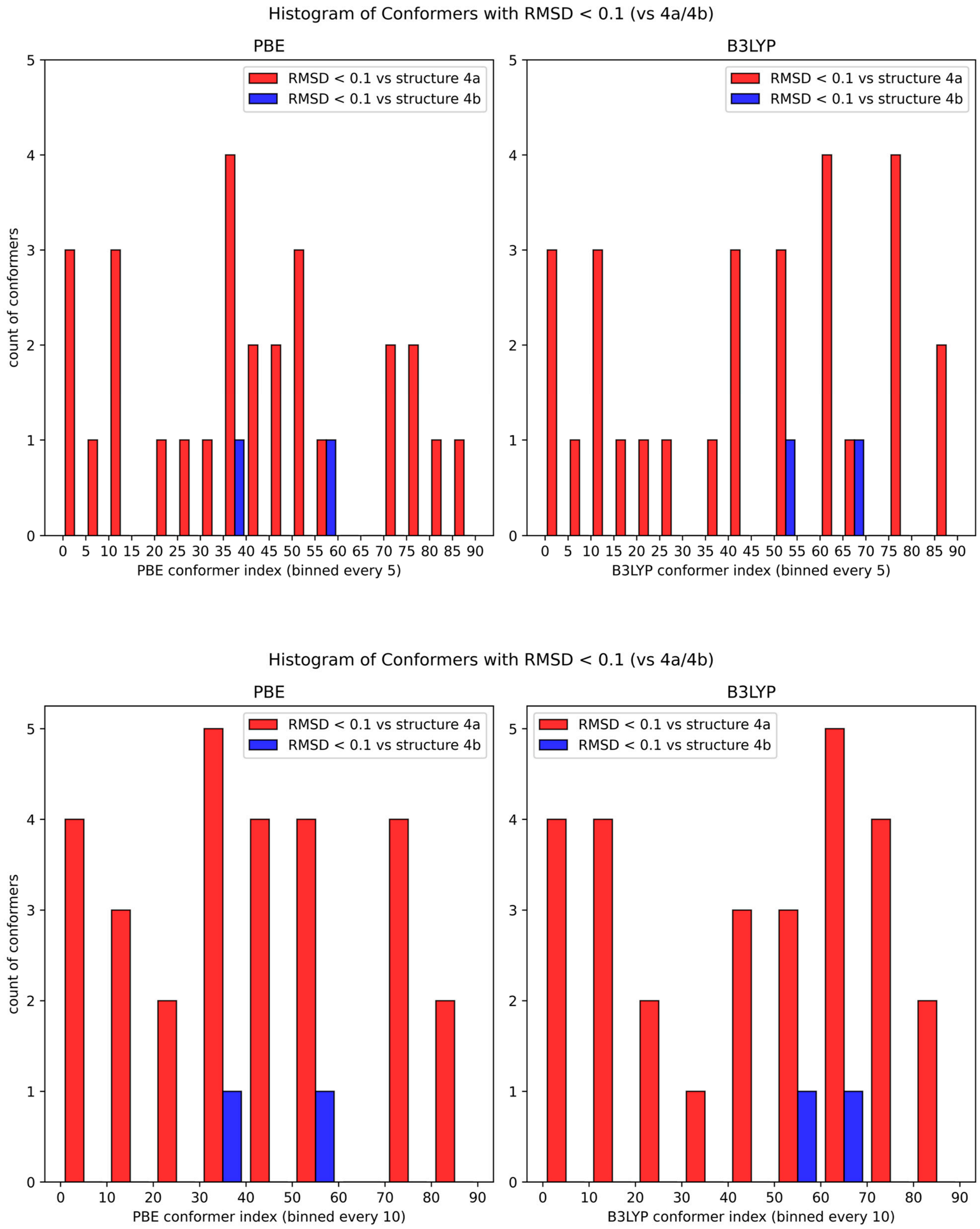

Figure S3. Distribution of the number of conformers exhibiting an RMSD of < 0.1 Å vs the structures displayed in Figure 4a and 4b depending on their ranks at the PBE-D3BJ/def2-SVP and the B3LYP-D3BJ/def2-SVP@PBE-D3BJ/def2-SVP levels of theory, respectively. Top: bin size of five ranks; bottom: bin size of 10 ranks.

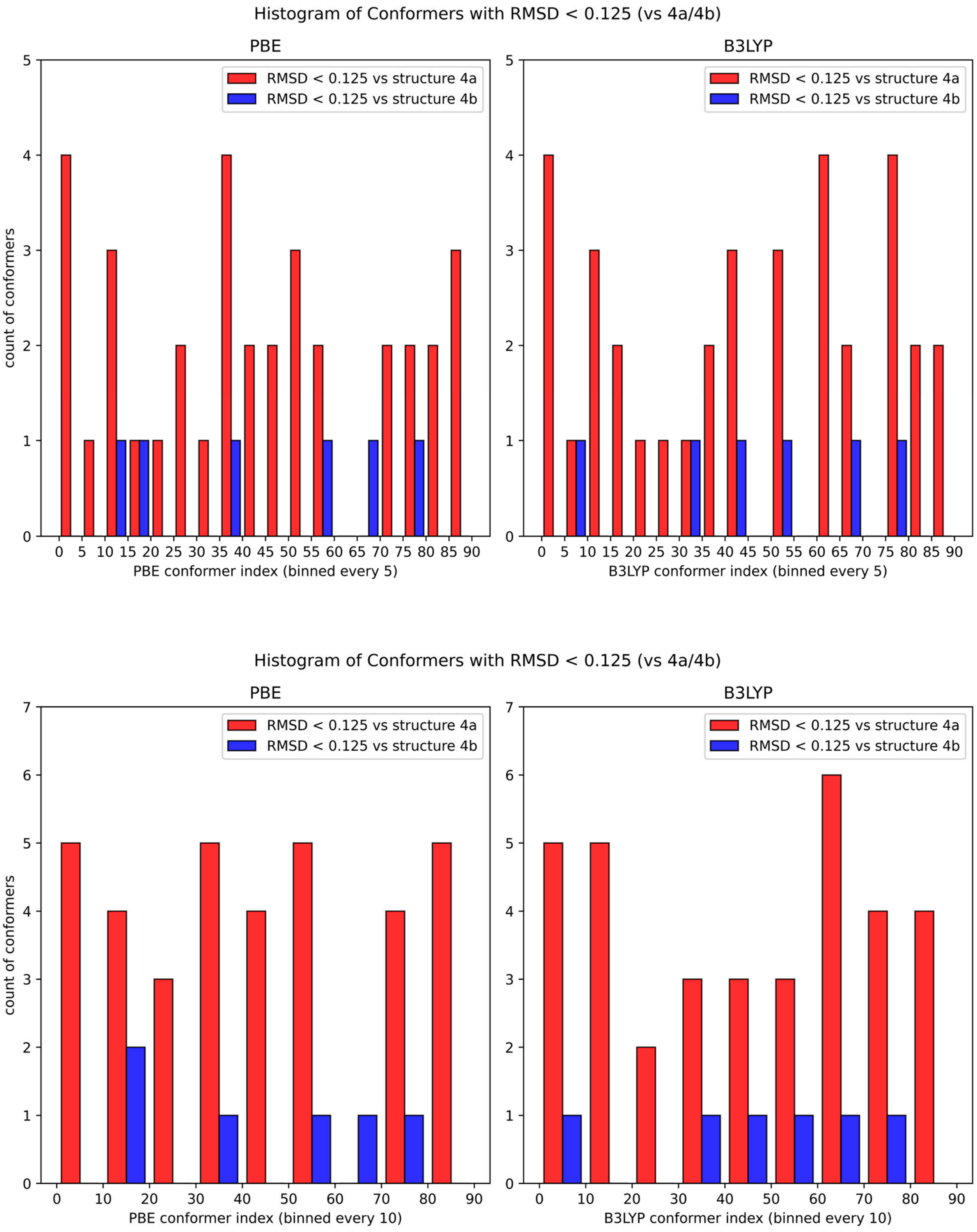

Figure S4. Distribution of the number of conformers exhibiting an RMSD of < 0.125 Å vs the structures displayed in Figure 4a and 4b depending on their ranks at the PBE-D3BJ/def2-SVP and the B3LYP-D3BJ/def2-SVP@PBE-D3BJ/def2-SVP levels of theory, respectively. Top: bin size of five ranks; bottom: bin size of 10 ranks.

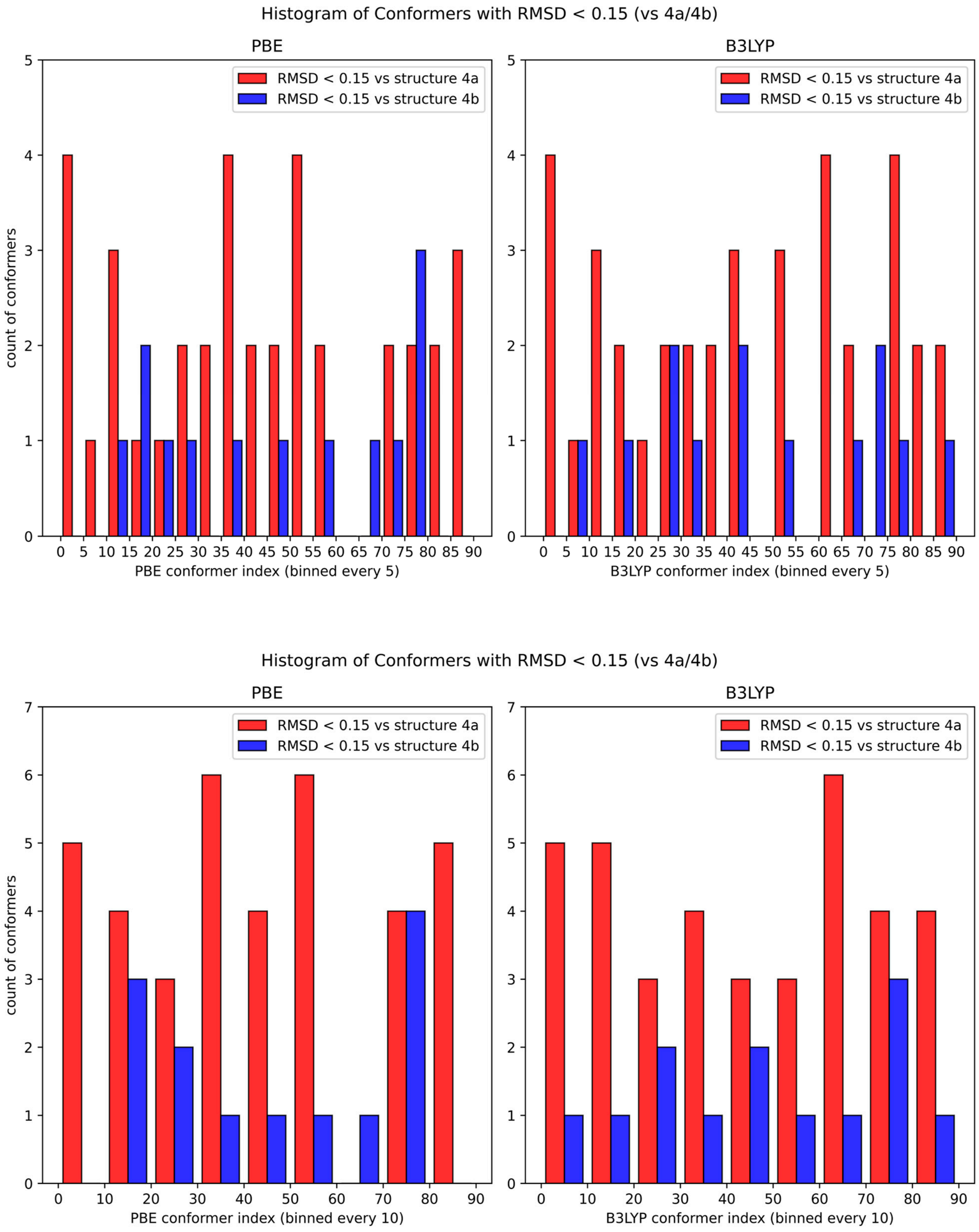

Figure S5: Distribution of the number of conformers exhibiting an RMSD of < 0.15 Å vs the structures displayed in Figure 4a and 4b depending on their ranks at the PBE-D3BJ/def2-SVP and the B3LYP-D3BJ/def2-SVP@PBE-D3BJ/def2-SVP levels of theory, respectively. Top: bin size of five ranks; bottom: bin size of 10 ranks.

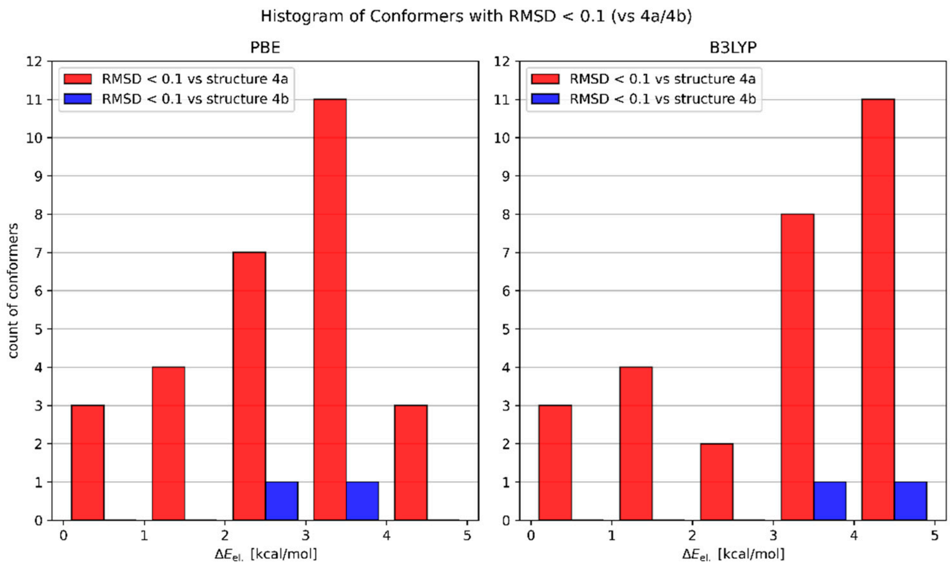
Histogram of Conformers with RMSD < 0.1 (vs 4a/4b)
PBE
B3LYP
RMSD < 0.1 vs structure 4a
RMSD < 0.1 vs structure 4b
count of conformers
ΔE_el. [kcal/mol]

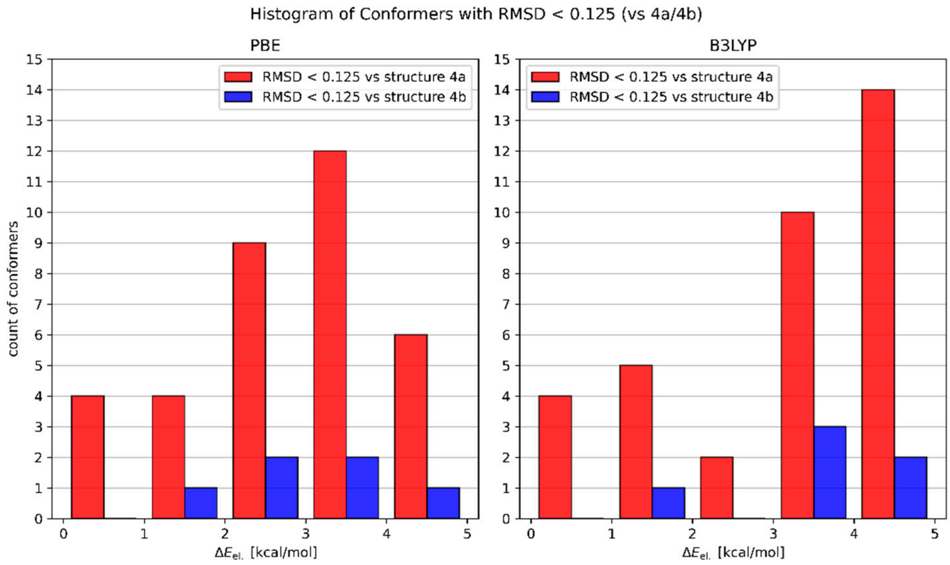
Histogram of Conformers with RMSD < 0.125 (vs 4a/4b)
PBE
B3LYP
RMSD < 0.125 vs structure 4a
RMSD < 0.125 vs structure 4b
count of conformers
ΔE_el. [kcal/mol]

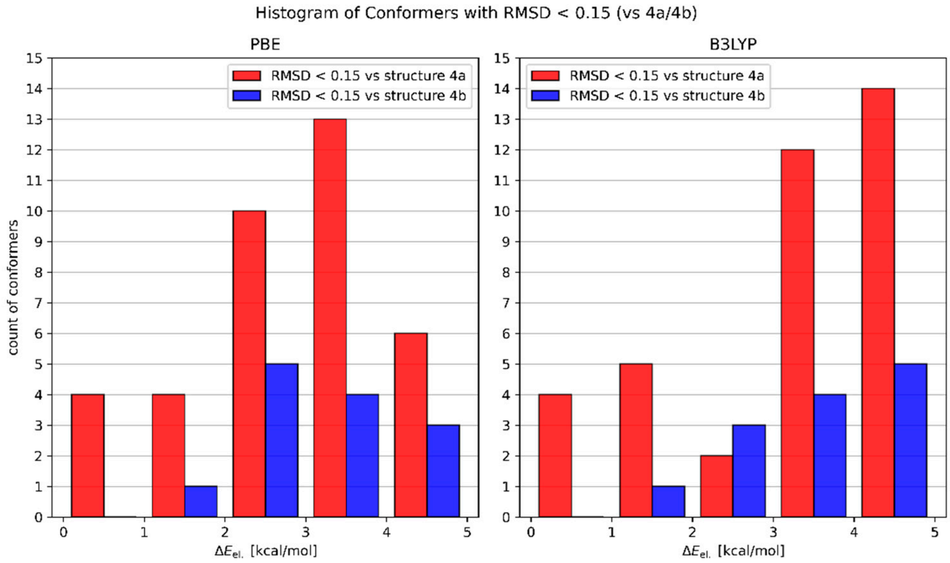
Histogram of Conformers with RMSD < 0.15 (vs 4a/4b)
PBE
B3LYP
RMSD < 0.15 vs structure 4a
RMSD < 0.15 vs structure 4b
count of conformers
ΔE_el. [kcal/mol]

Figure S6. Distribution of the number of conformers exhibiting an RMSD of < 0.1 (top), 0.125 (middle) and 0.15 Å (bottom) vs the structures displayed in Figure 4a and 4b depending on their relative electronic energies (vs the most stable conformer) at the PBE-D3BJ/def2-SVP and the B3LYP-D3BJ/def2-SVP@PBE-D3BJ/def2-SVP level of theory, respectively). The bin size was chosen to be 1 kcal/mol.

Rendered Images of Selected Conformers and Comparison to the Structures Depicted in Figure 4a and 4b

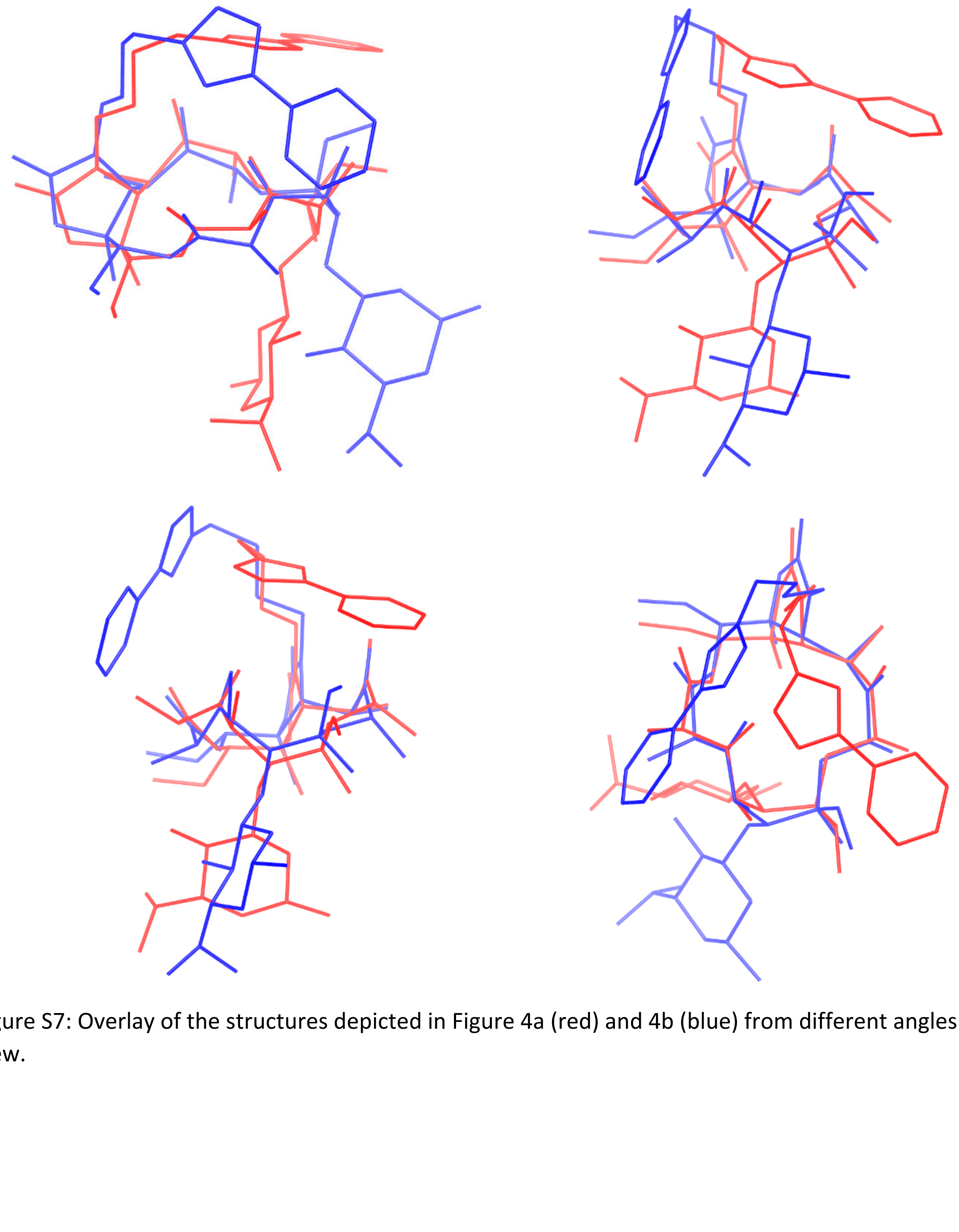

Figure S7: Overlay of the structures depicted in Figure 4a (red) and 4b (blue) from different angles of view.

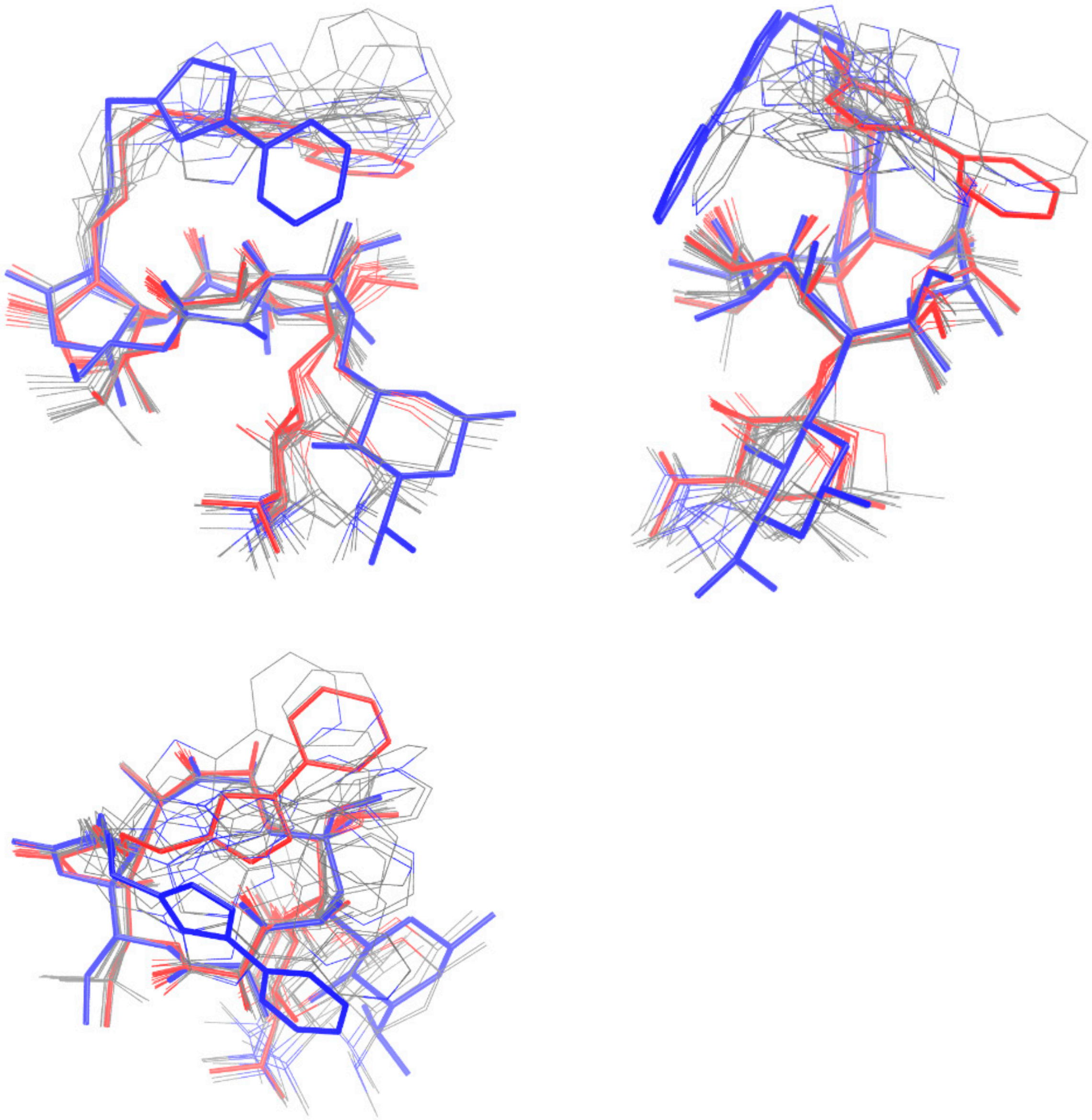

Figure S8. Overlay of the first 20 conformers (ranked according to the B3LYP-D3BJ/def2-SVP@PBE-D3BJ/def2-SVP level of theory) and the structures depicted in Figure 4a (red) and 4b (blue) from different angles of view.

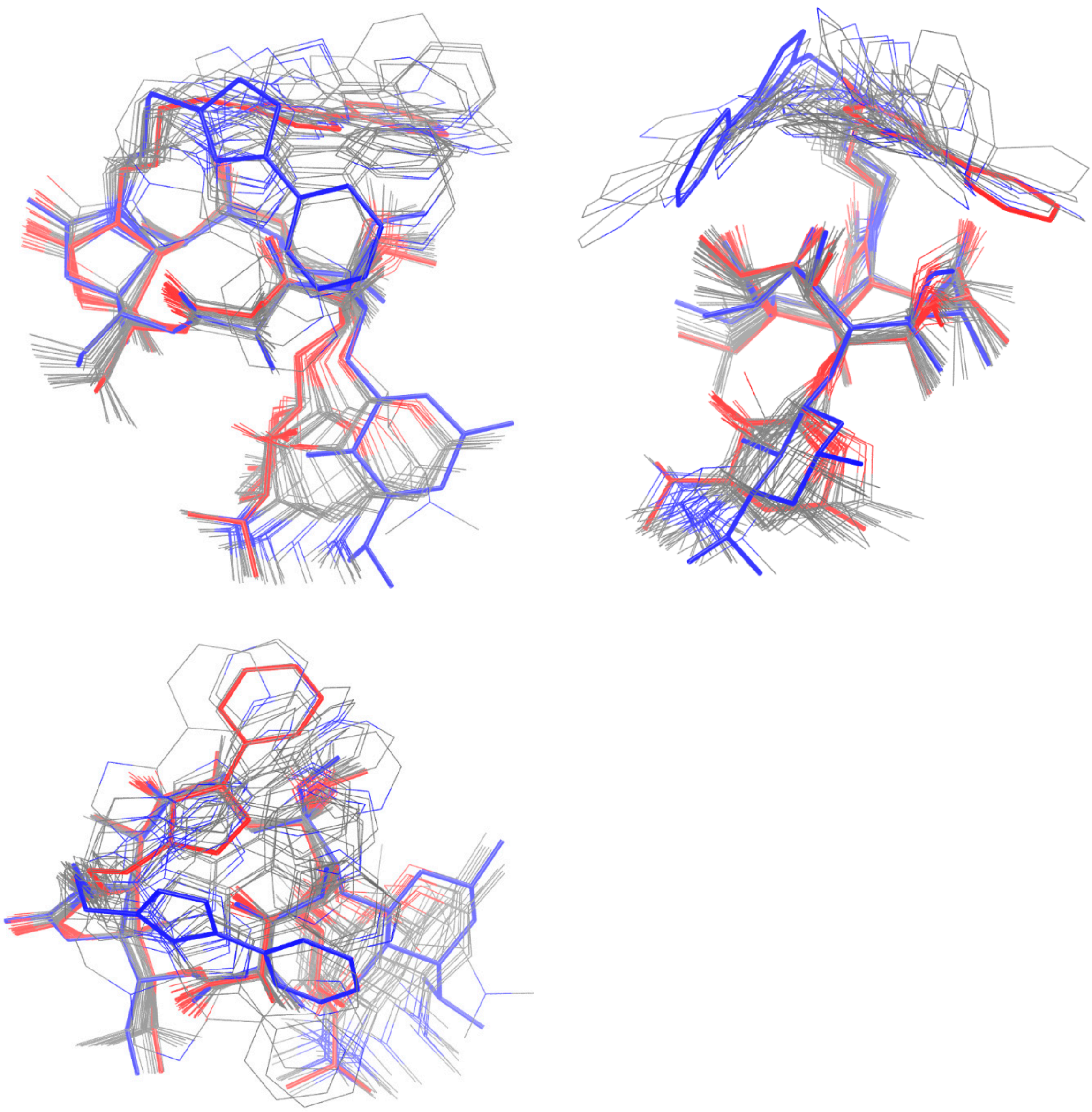

Figure S9. Overlay of the first 40 conformers (ranked according to the B3LYP-D3BJ/def2-SVP@PBE-D3BJ/def2-SVP level of theory) and the structures depicted in Figure 4a (red) and 4b (blue) from different angles of view.

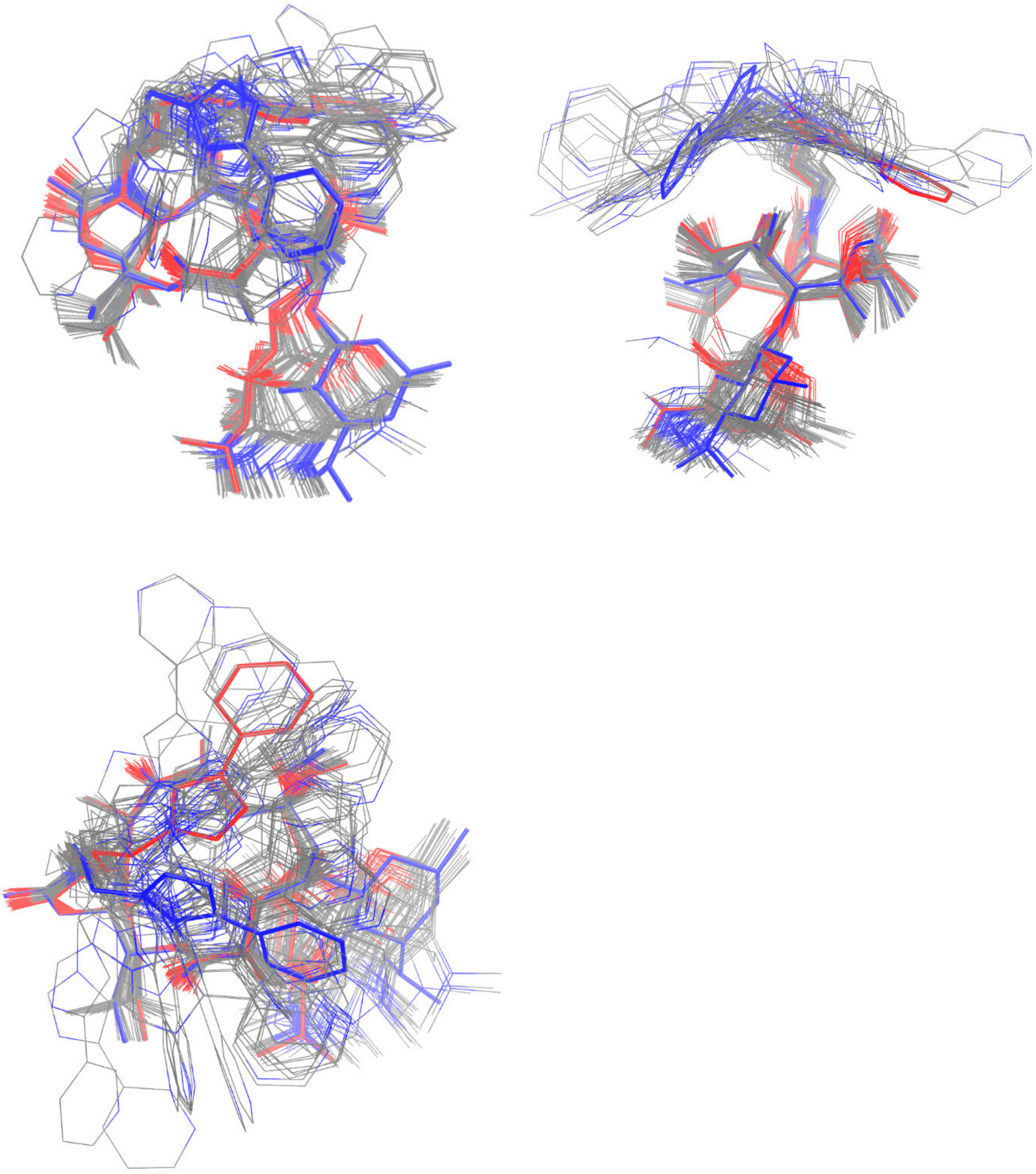

Figure S10. Overlay of the first 90 conformers (ranked according to the B3LYP-D3BJ/def2-SVP@PBE-D3BJ/def2-SVP level of theory) and the structures depicted in Figure 4a (red) and 4b (blue) from different angles of view.

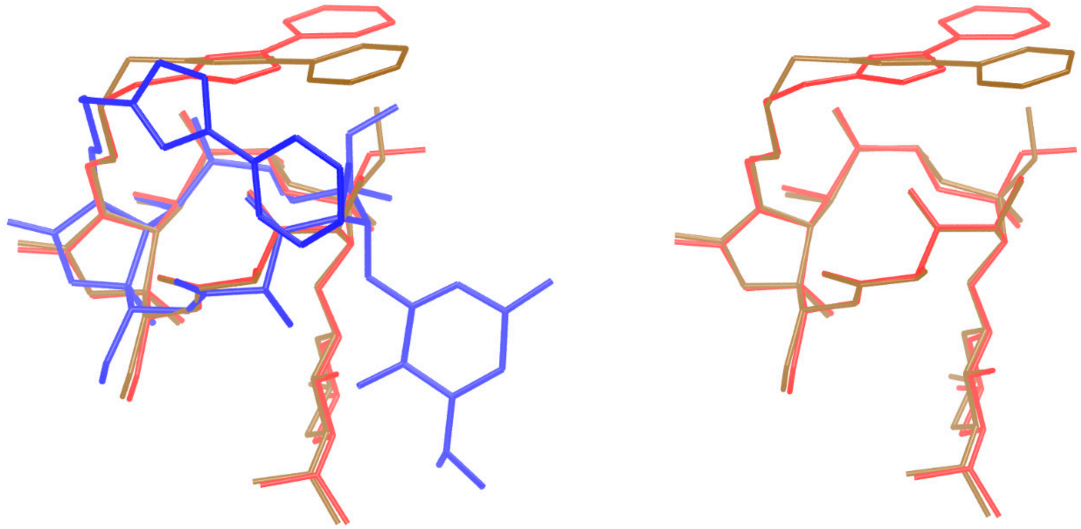

Figure S11. Overlay of the conformer with rank 0 (ochre; ranked according to the B3LYP-D3BJ/def2-SVP@PBE-D3BJ/def2-SVP level of theory) and the structures depicted in Figure 4a (red) and 4b (blue).

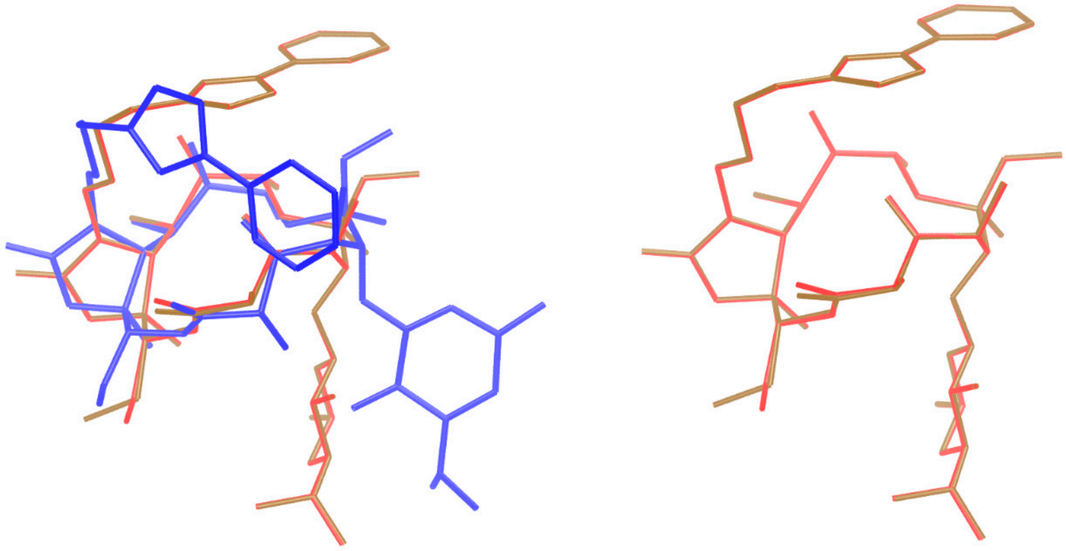

Figure S12: Overlay of the conformer with rank 1 (ochre; ranked according to the B3LYP-D3BJ/def2-SVP@PBE-D3BJ/def2-SVP level of theory) and the structures depicted in Figure 4a (red) and 4b (blue).

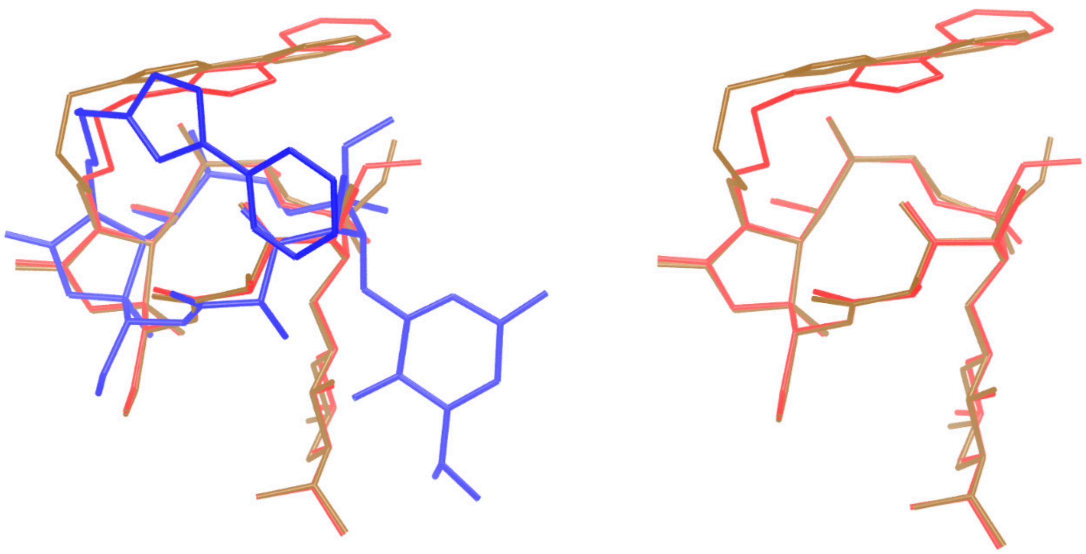

Figure S13. Overlay of the conformer with rank 2 (ochre; ranked according to the B3LYP-D3BJ/def2-SVP@PBE-D3BJ/def2-SVP level of theory) and the structures depicted in Figure 4a (red) and 4b (blue).

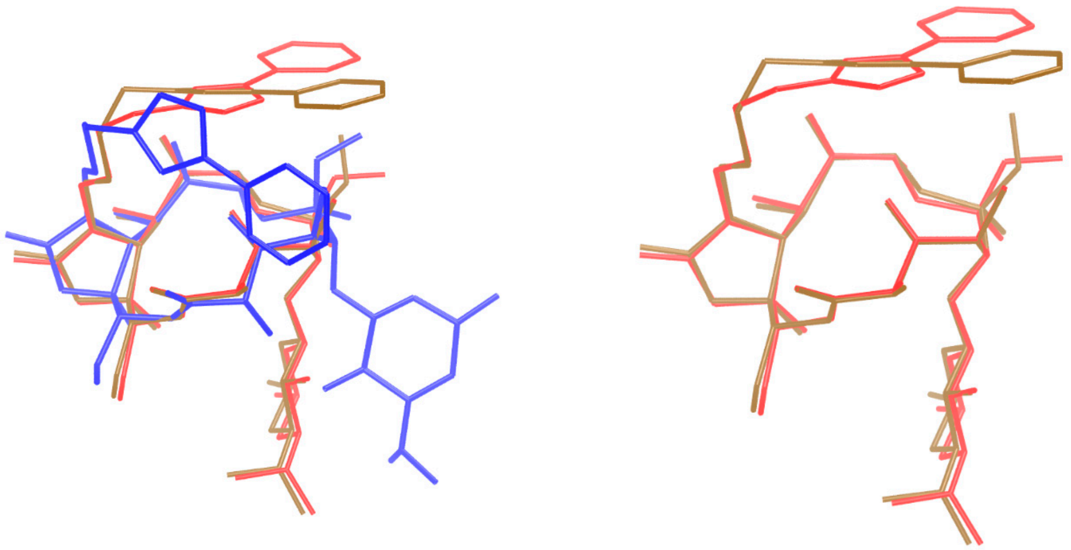

Figure S14. Overlay of the conformer with rank 3 (ochre; ranked according to the B3LYP-D3BJ/def2-SVP@PBE-D3BJ/def2-SVP level of theory) and the structures depicted in Figure 4a (red) and 4b (blue).

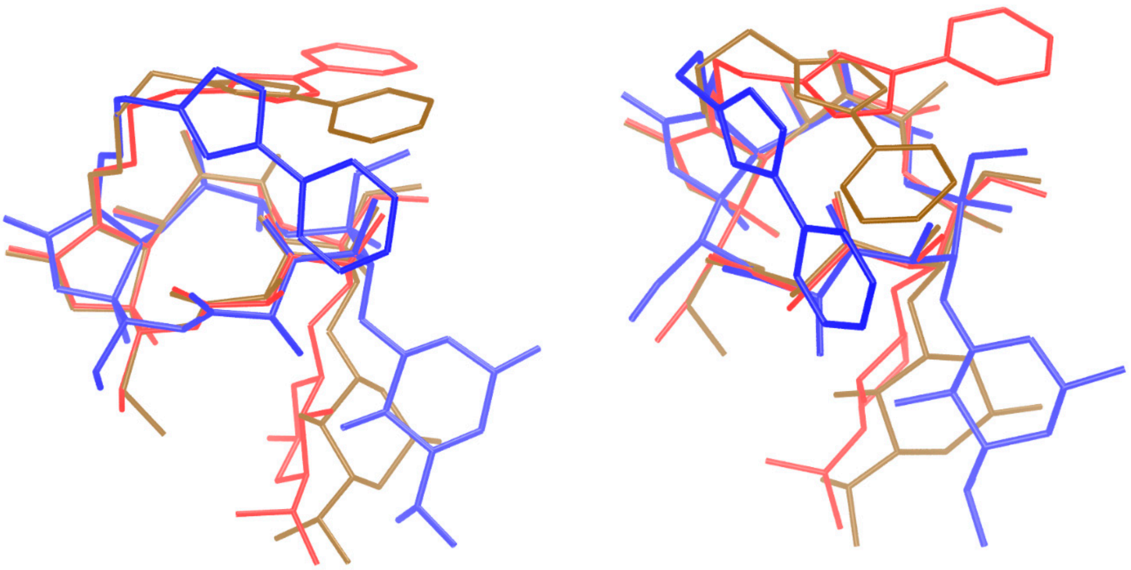

Figure S15. Overlay of the conformer with rank 4 (ochre; ranked according to the B3LYP-D3BJ/def2-SVP@PBE-D3BJ/def2-SVP level of theory) and the structures depicted in Figure 4a (red) and 4b (blue).

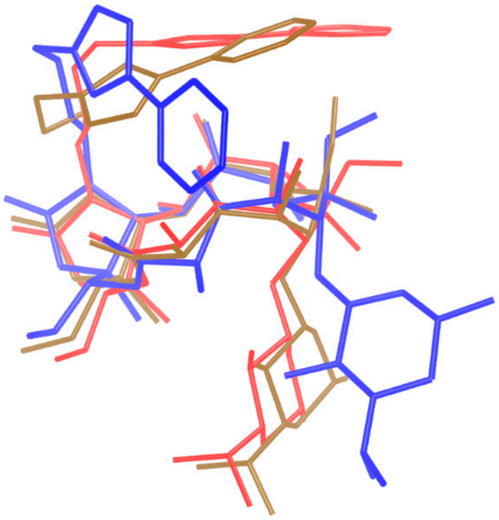

Figure S16. Overlay of the conformer with rank 5 (ochre; ranked according to the B3LYP-D3BJ/def2-SVP@PBE-D3BJ/def2-SVP level of theory) and the structures depicted in Figure 4a (red) and 4b (blue).

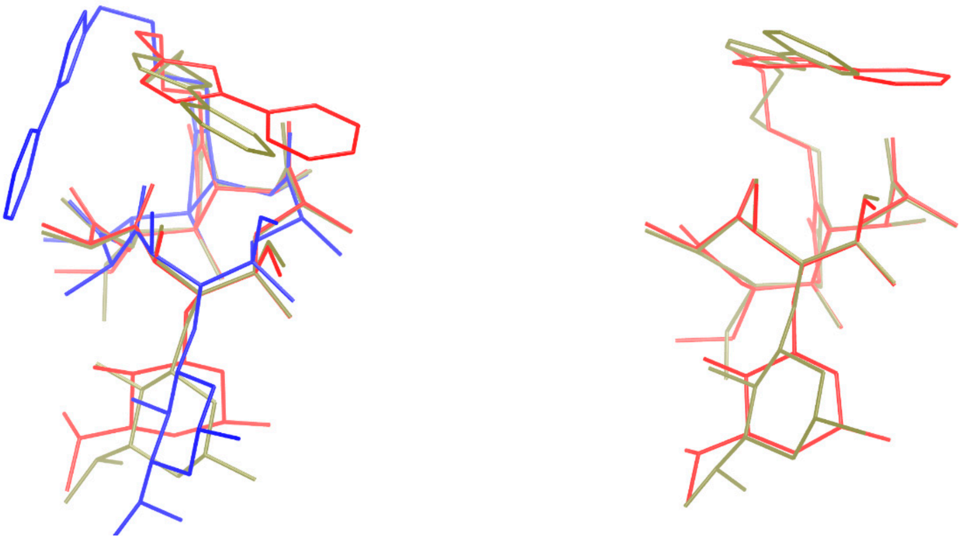

Figure S17: Overlay of the conformer with rank 6 (ochre; ranked according to the B3LYP-D3BJ/def2-SVP@PBE-D3BJ/def2-SVP level of theory) and the structures depicted in Figure 4a (red) and 4b (blue).

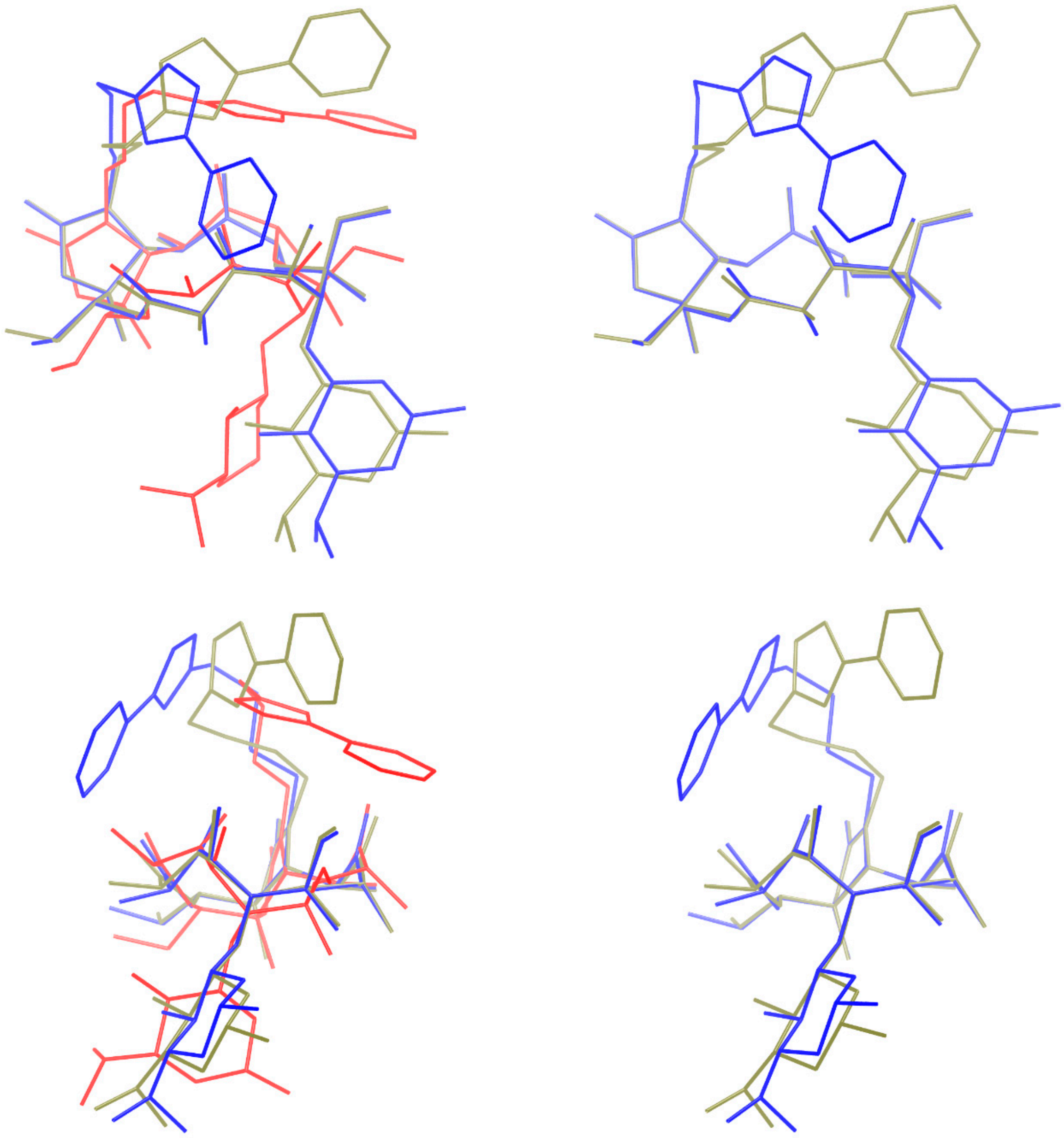

Figure S18: Overlay of the conformer with rank 8 (ochre; ranked according to the B3LYP-D3BJ/def2-SVP@PBE-D3BJ/def2-SVP level of theory) and the structures depicted in Figure 4a (red) and 4b (blue).

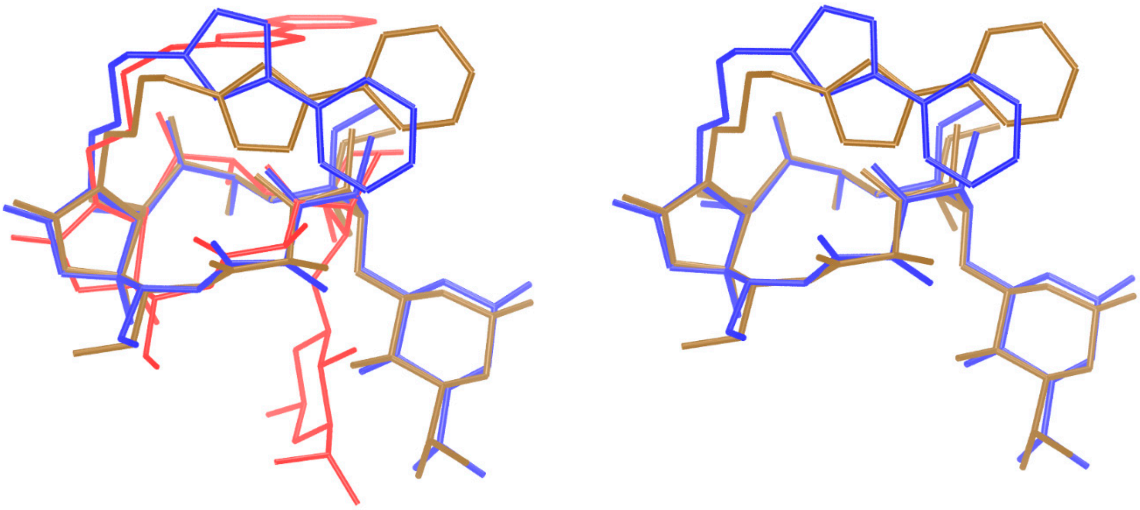

Figure S19. Overlay of the conformer with rank 9 (ochre; ranked according to the B3LYP-D3BJ/def2-SVP@PBE-D3BJ/def2-SVP level of theory) and the structures depicted in Figure 4a (red) and 4b (blue).

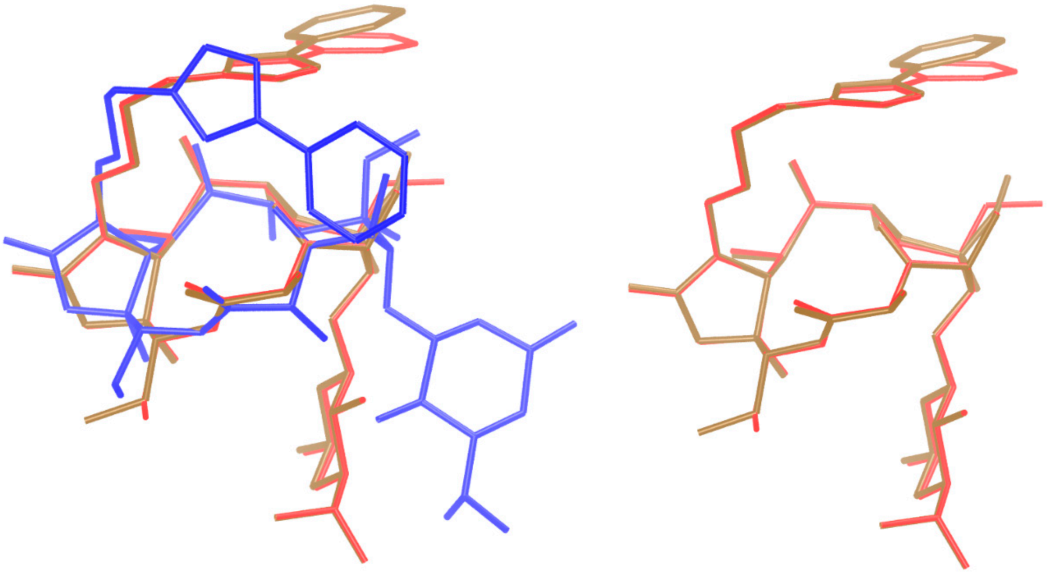

Figure S20. Overlay of the conformer with rank 12 (ochre; ranked according to the B3LYP-D3BJ/def2-SVP@PBE-D3BJ/def2-SVP level of theory) and the structures depicted in Figure 4a (red) and 4b (blue).

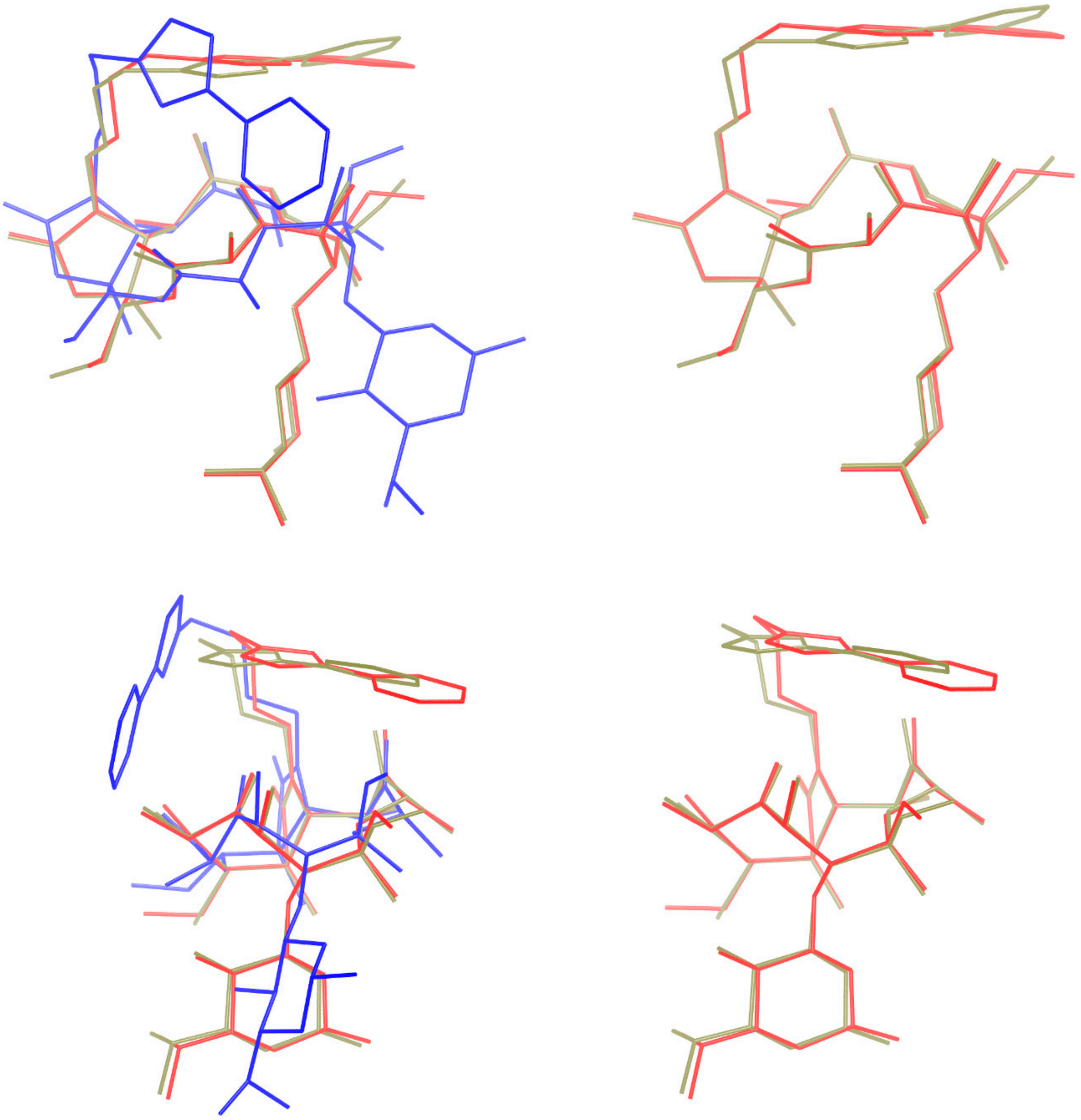

Figure S21. Overlay of the conformer with rank 13 (ochre; ranked according to the B3LYP-D3BJ/def2-SVP@PBE-D3BJ/def2-SVP level of theory) and the structures depicted in Figure 4a (red) and 4b (blue).

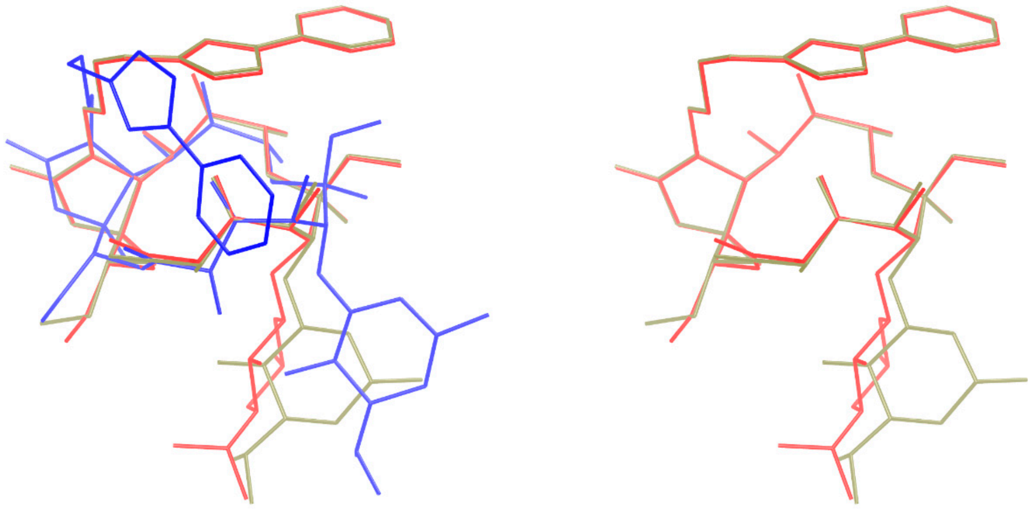

Figure S22. Overlay of the conformer with rank 14 (ochre; ranked according to the B3LYP-D3BJ/def2-SVP@PBE-D3BJ/def2-SVP level of theory) and the structures depicted in Figure 4a (red) and 4b (blue).

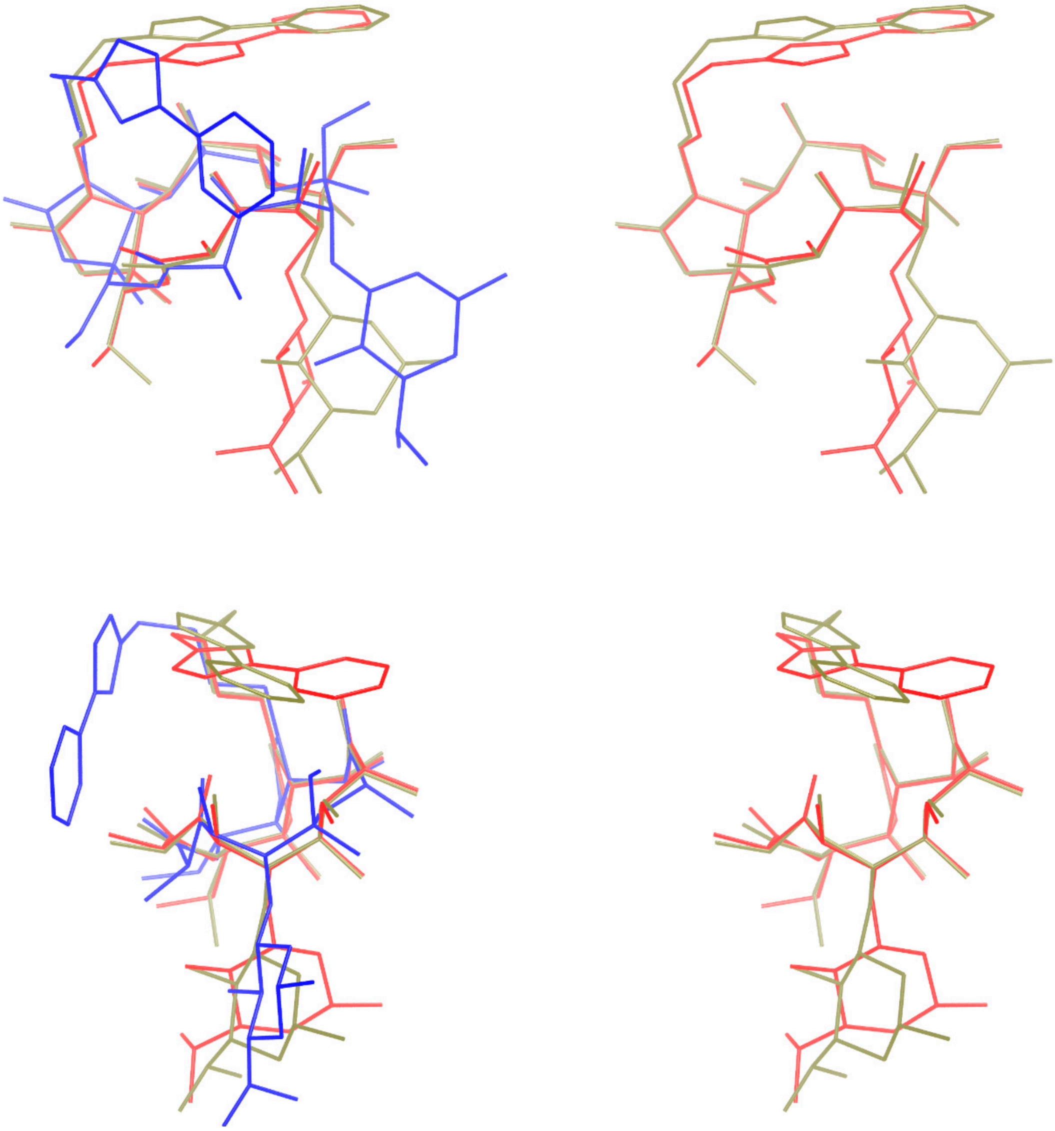

Figure S23. Overlay of the conformer with rank 15 (ochre; ranked according to the B3LYP-D3BJ/def2-SVP@PBE-D3BJ/def2-SVP level of theory) and the structures depicted in Figure 4a (red) and 4b (blue).

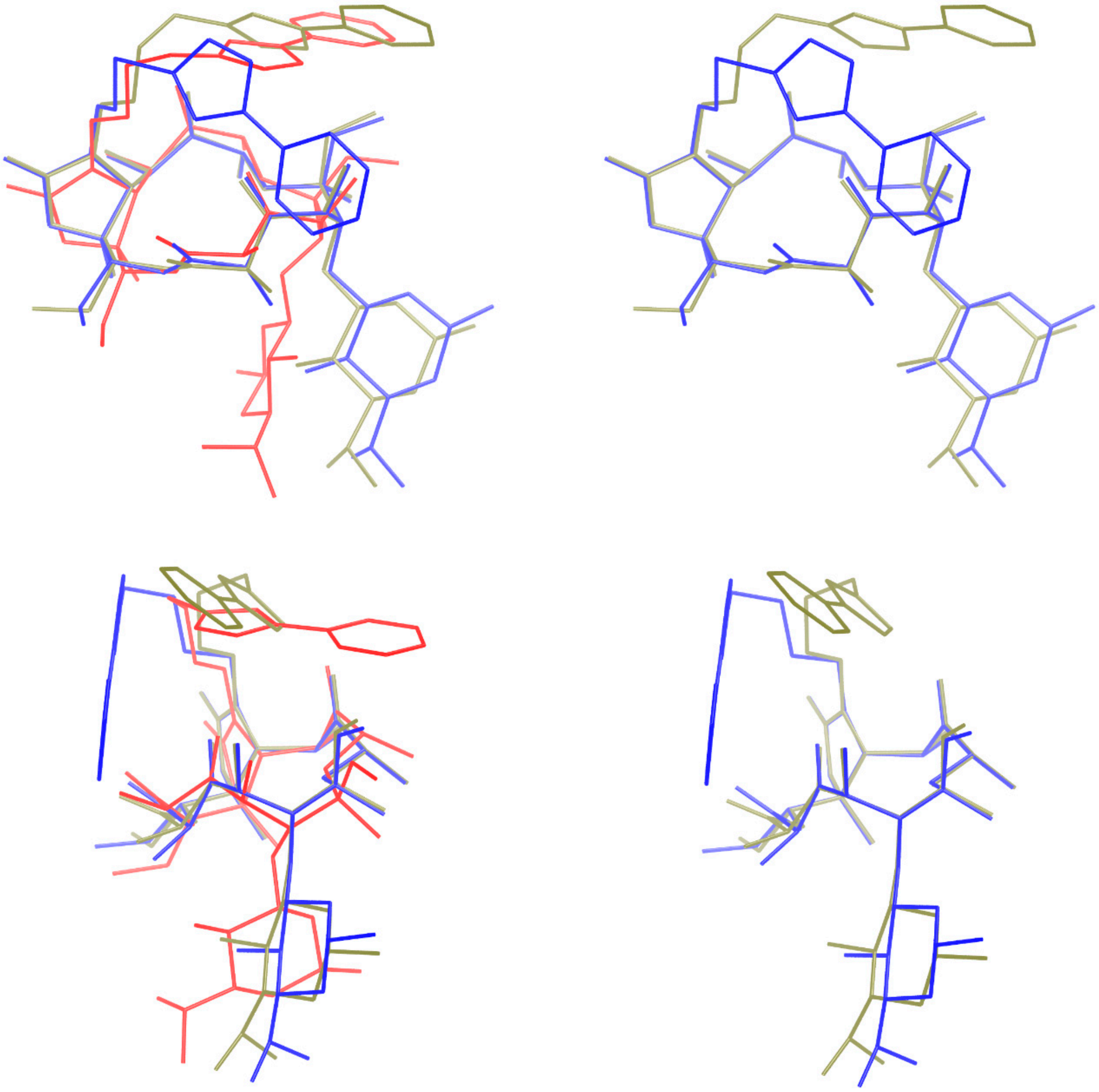

Figure S24. Overlay of the conformer with rank 17 (ochre; ranked according to the B3LYP-D3BJ/def2-SVP@PBE-D3BJ/def2-SVP level of theory) and the structures depicted in Figure 4a (red) and 4b (blue).

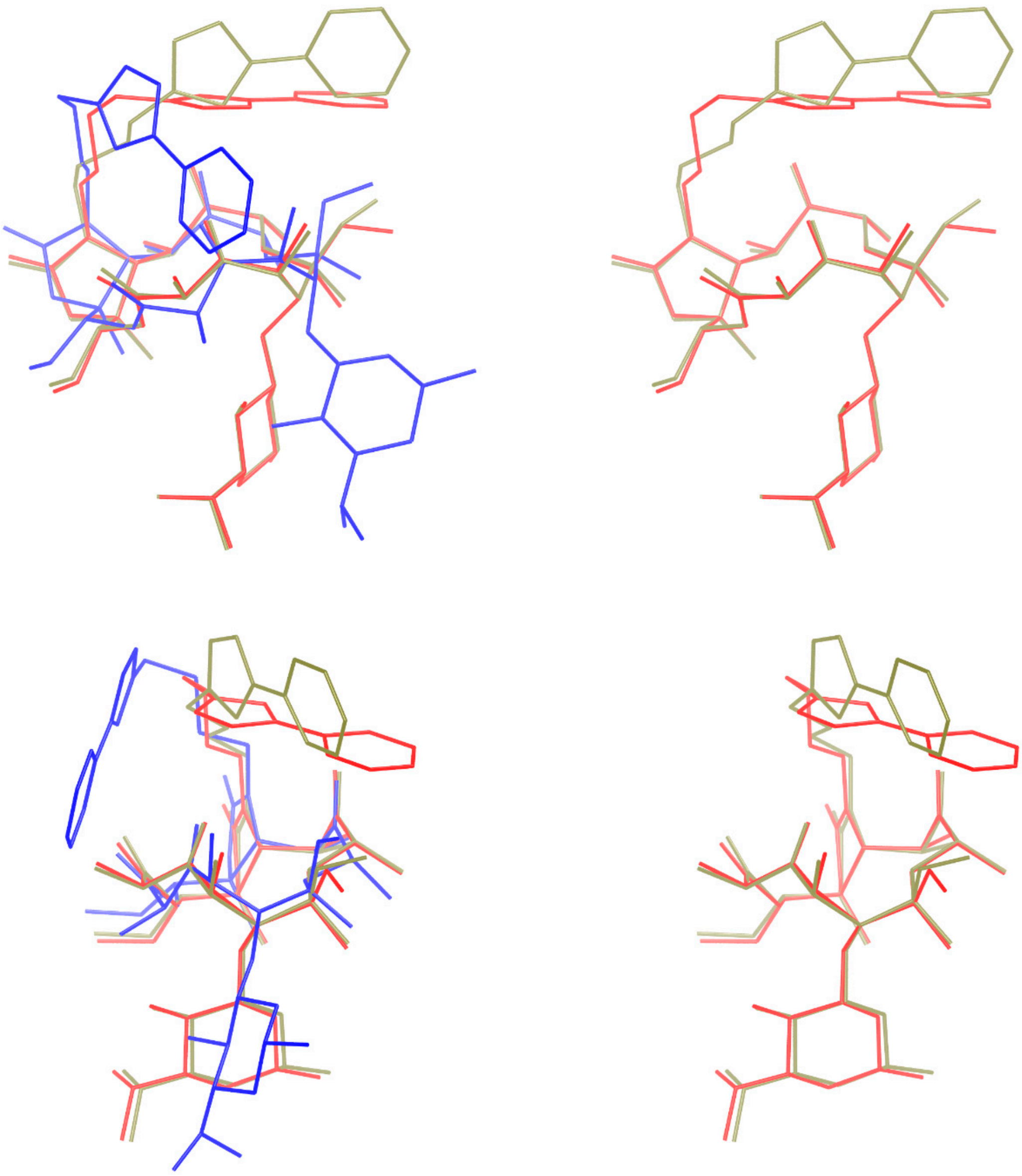

Figure S25. Overlay of the conformer with rank 18 (ochre; ranked according to the B3LYP-D3BJ/def2-SVP@PBE-D3BJ/def2-SVP level of theory) and the structures depicted in Figure 4a (red) and 4b (blue).

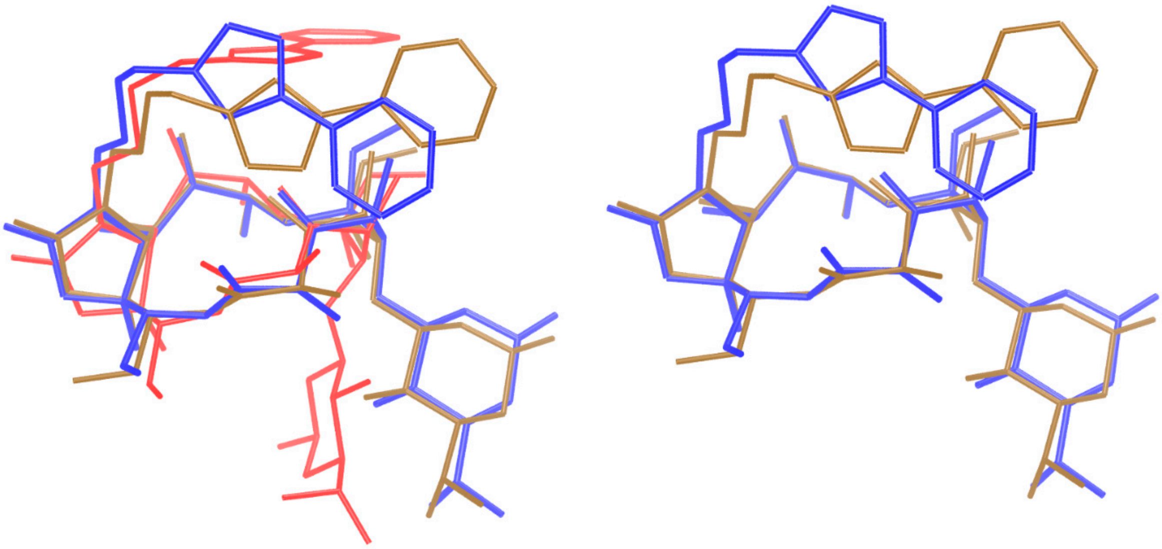

Figure S26. Overlay of the conformer with rank 19 (ochre; ranked according to the B3LYP-D3BJ/def2-SVP@PBE-D3BJ/def2-SVP level of theory) and the structures depicted in Figure 4a (red) and 4b (blue).

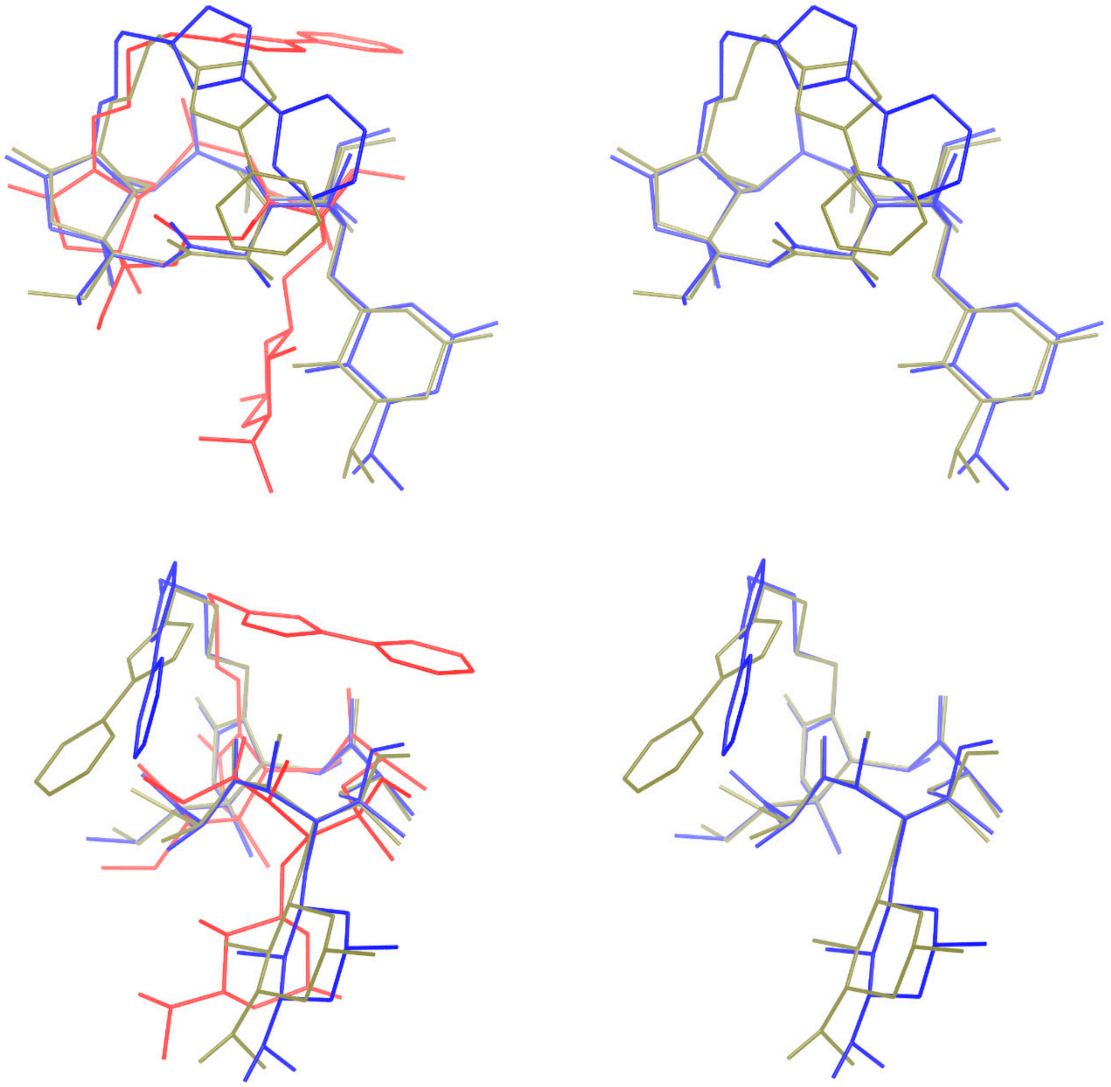

Figure S27. Overlay of the conformer with rank 34 (ochre; ranked according to the B3LYP-D3BJ/def2-SVP@PBE-D3BJ/def2-SVP level of theory) and the structures depicted in Figure 4a (red) and 4b (blue).

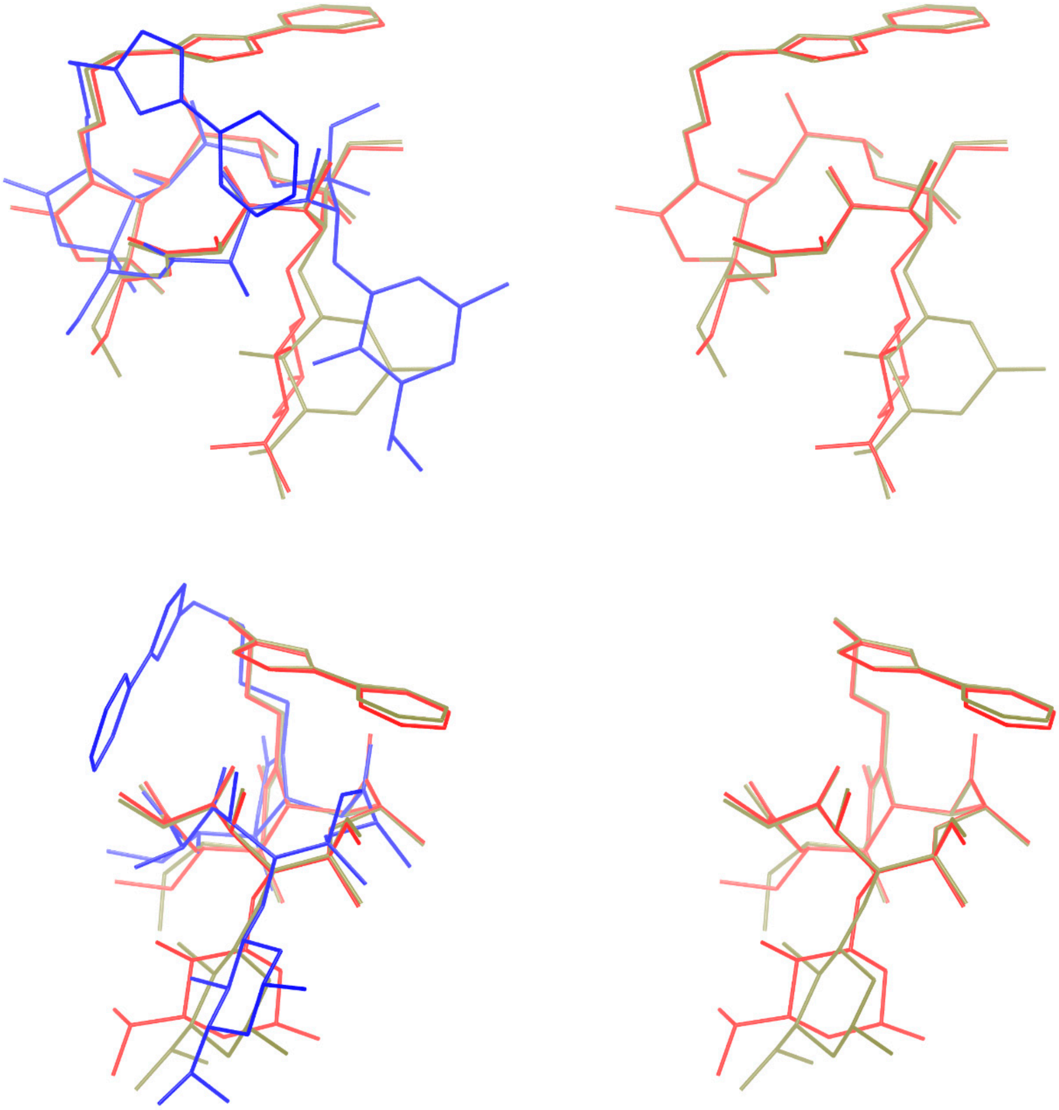

Figure S28. Overlay of the conformer with rank 35 (ochre; ranked according to the B3LYP-D3BJ/def2-SVP@PBE-D3BJ/def2-SVP level of theory) and the structures depicted in Figure 4a (red) and 4b (blue).

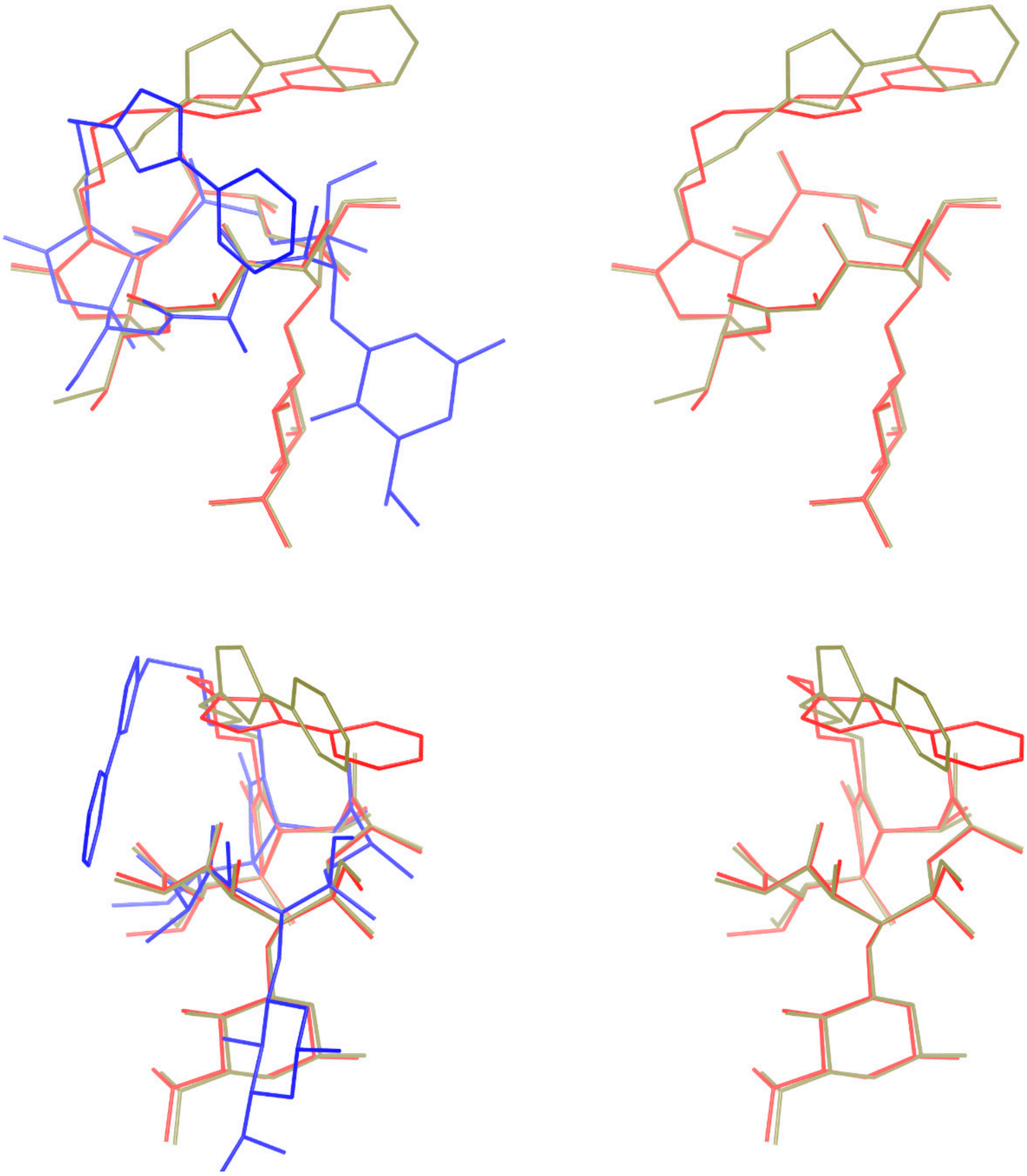

Figure S29. Overlay of the conformer with rank 40 (ochre; ranked according to the B3LYP-D3BJ/def2-SVP@PBE-D3BJ/def2-SVP level of theory) and the structures depicted in Figure 4a (red) and 4b (blue).

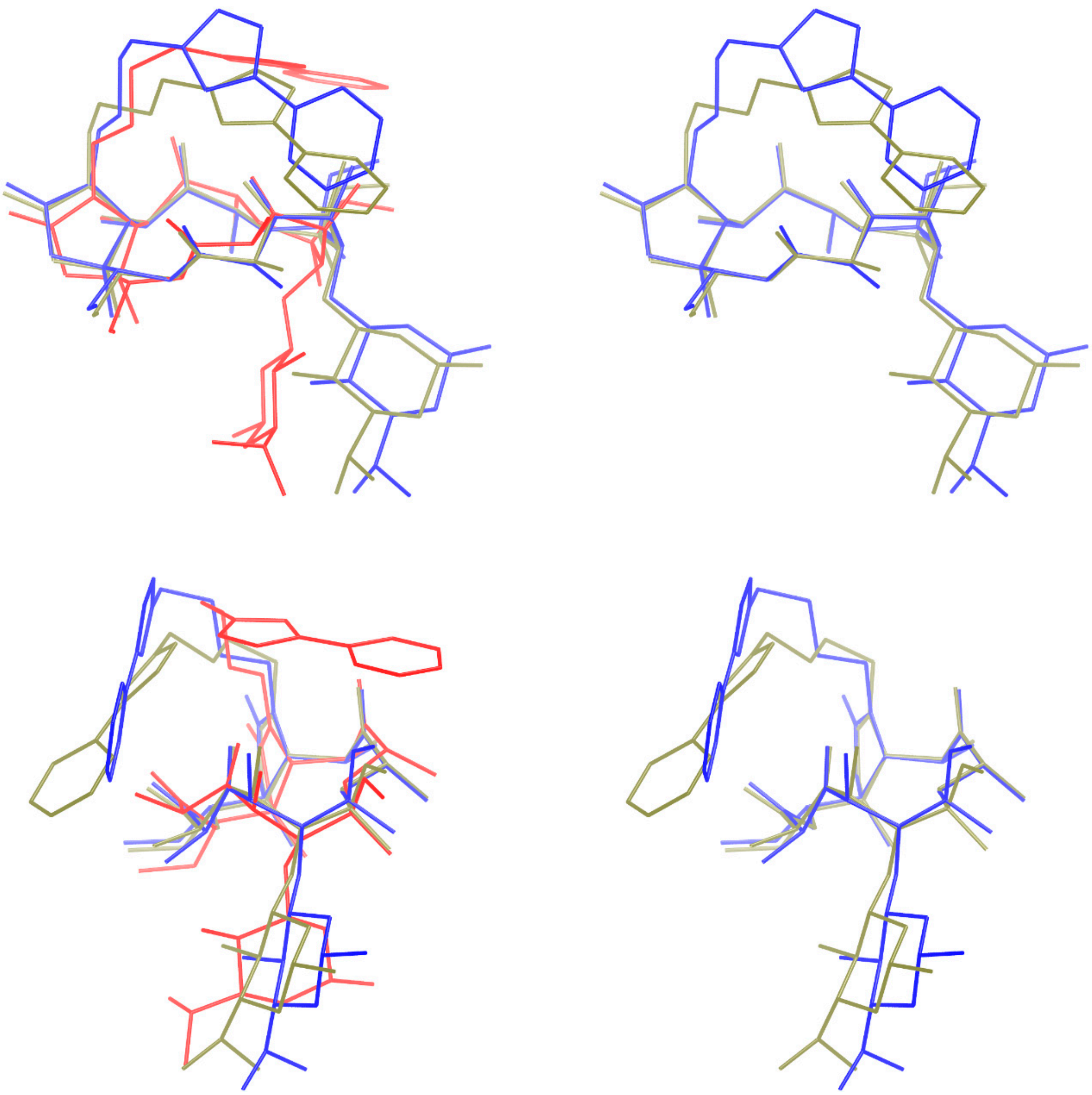

Figure S30. Overlay of the conformer with rank 41 (ochre; ranked according to the B3LYP-D3BJ/def2-SVP@PBE-D3BJ/def2-SVP level of theory) and the structures depicted in Figure 4a (red) and 4b (blue).

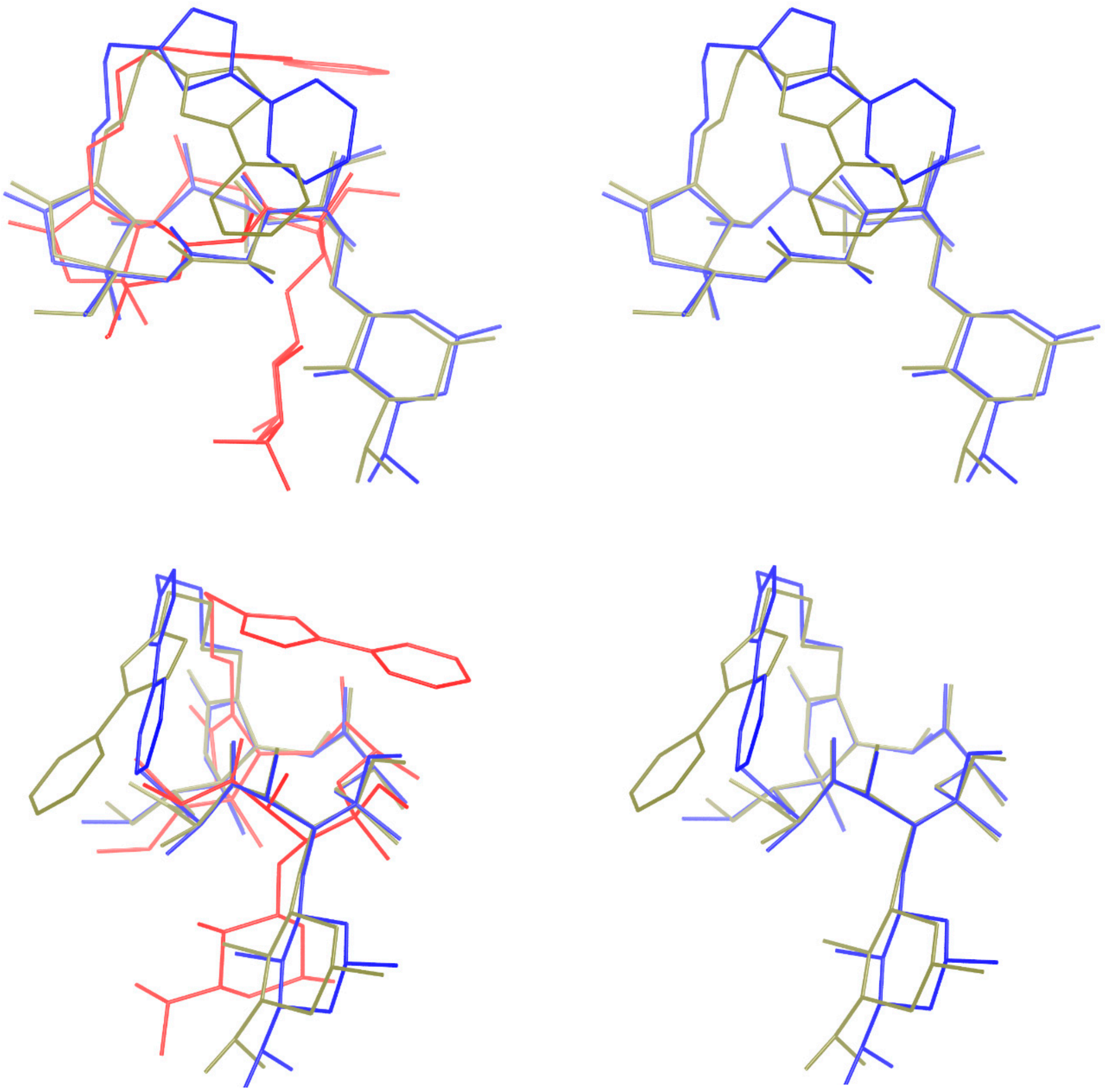

Figure S31. Overlay of the conformer with rank 44 (ochre; ranked according to the B3LYP-D3BJ/def2-SVP@PBE-D3BJ/def2-SVP level of theory) and the structures depicted in Figure 4a (red) and 4b (blue).

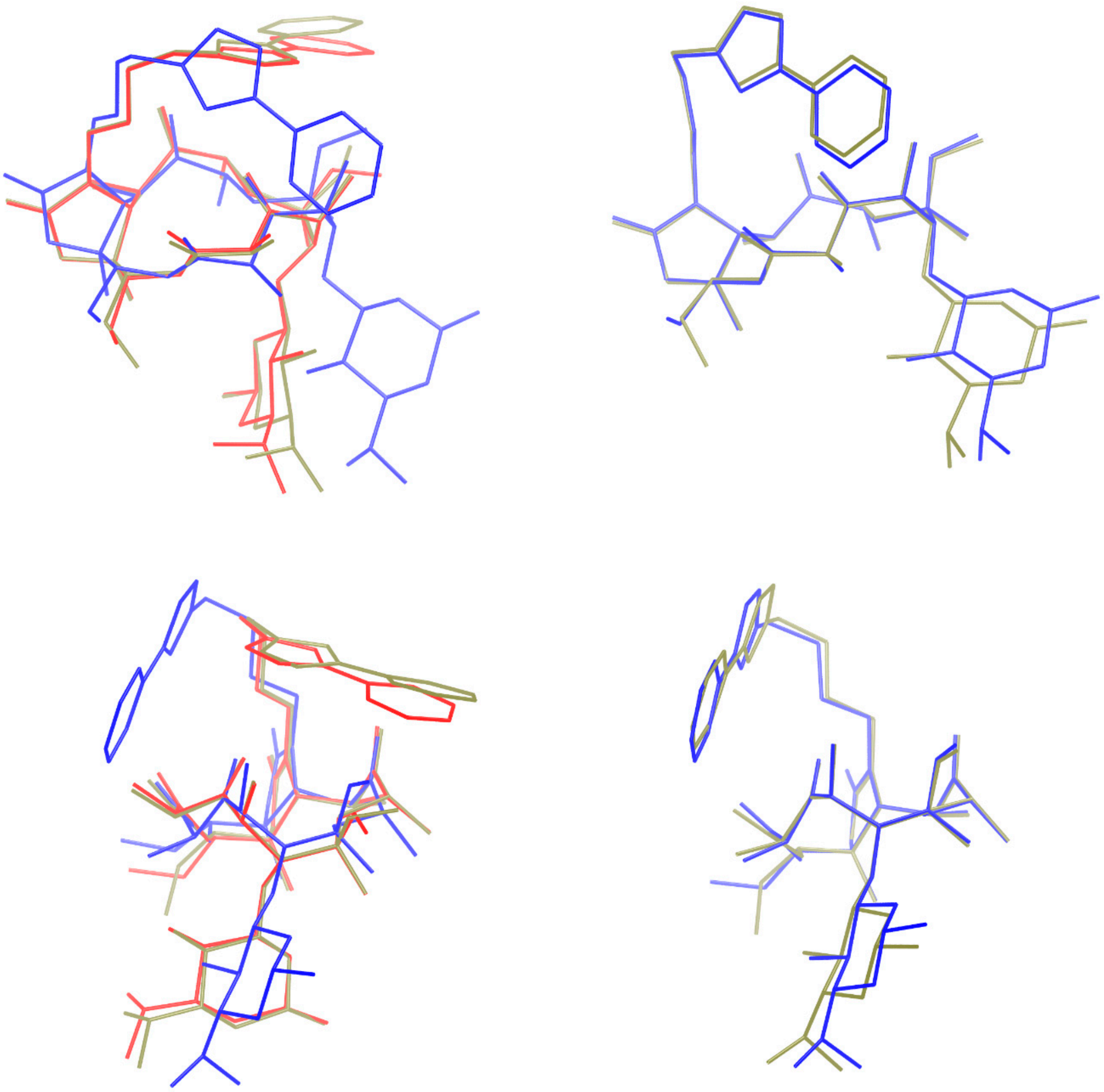

Figure S32: Overlay of the conformer with rank 50 (ochre; ranked according to the B3LYP-D3BJ/def2-SVP@PBE-D3BJ/def2-SVP level of theory) and the structures depicted in Figure 4a (red) and 4b (blue).

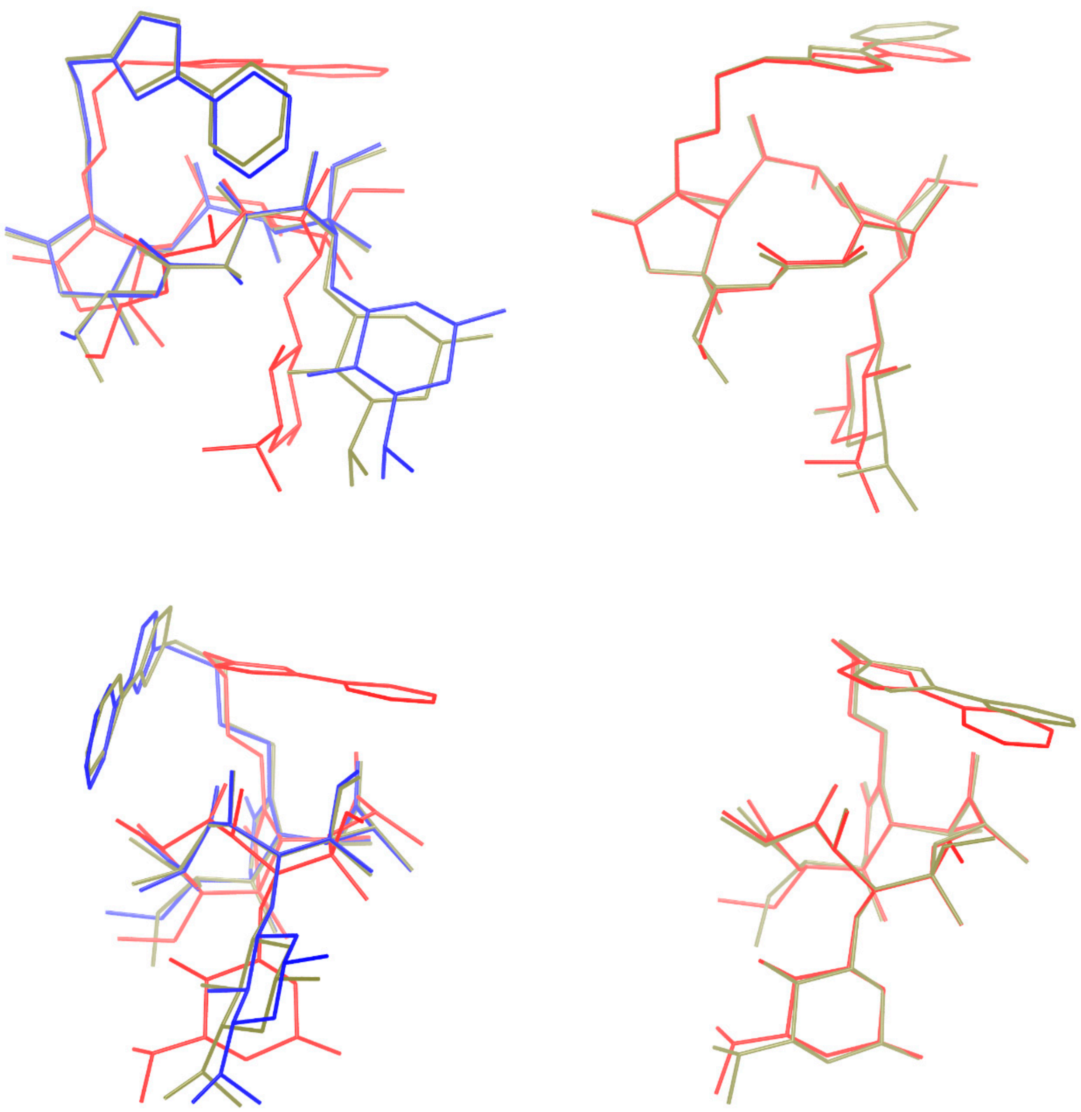

Figure S33. Overlay of the conformer with rank 52 (ochre; ranked according to the B3LYP-D3BJ/def2-SVP@PBE-D3BJ/def2-SVP level of theory) and the structures depicted in Figure 4a (red) and 4b (blue).

Effect of the Chosen Density Functional Approximation, Basis Set Size and Relative Permittivity on the Obtained Geometry

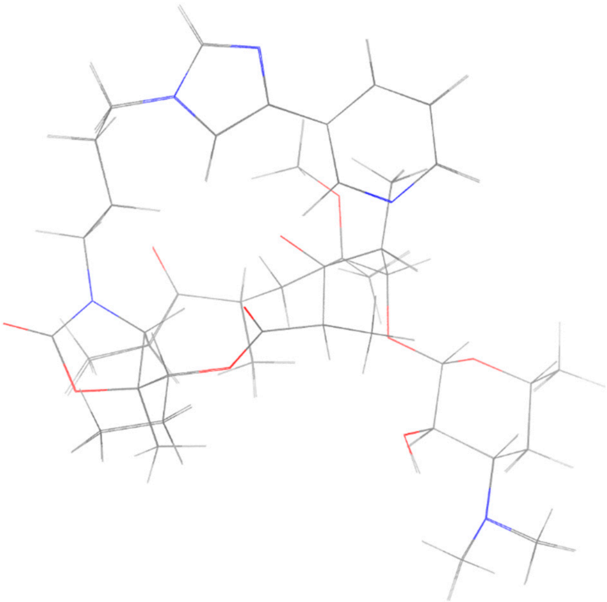

Figure S34. Overlay of the structures of conformer with rank 32 (ranked after the PBE-D3BJ/def2-SVP optimized energies obtained from the conformation analysis, see “Methods” in the main text) optimized in gas-phase at the PBE-D3BJ/def2-SVP and the B3LYP-D3BJ/def2-SVP levels of theory, respectively.

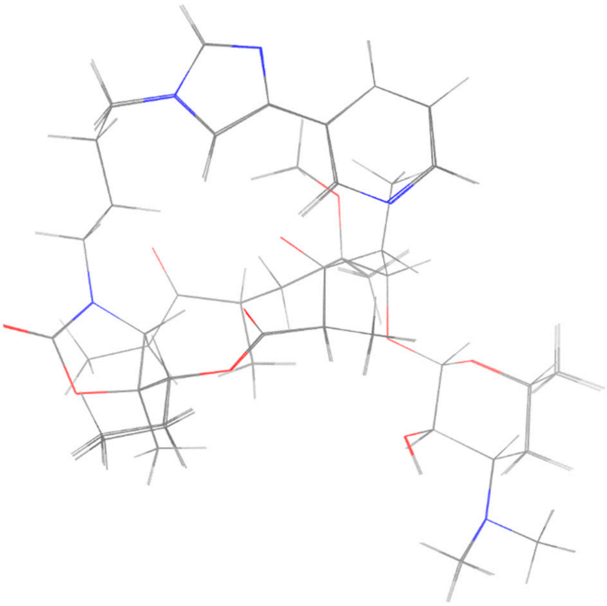

Figure S35. Overlay of the structures of conformer with rank 32 (ranked after the PBE-D3BJ/def2-SVP optimized energies obtained from the conformation analysis, see “Methods” in the main text) optimized at the PBE-D3BJ/def2-SVP level of theory in gas phase and employing the COSMO model at different relative permittivities (ε_r = 1.84, 2.38, 8.93, 24.55, 80.1 – corresponding to pentane, toluene, DCM, EtOH and water, respectively).

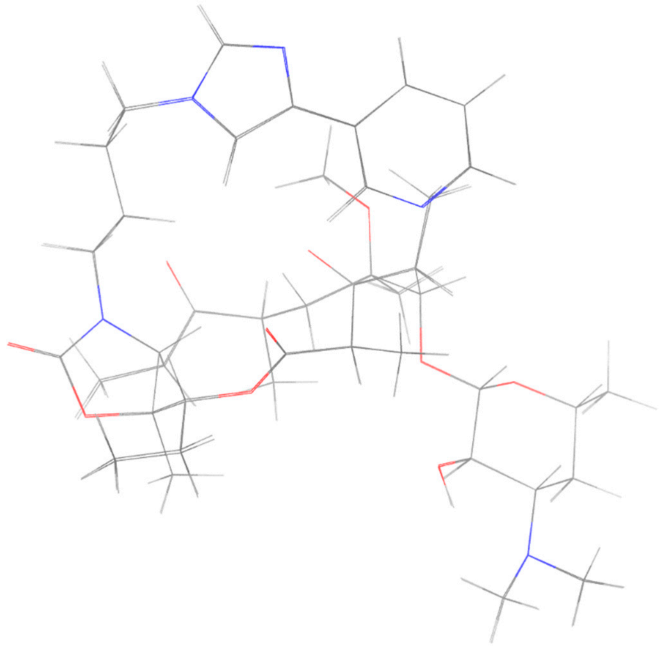

Figure S36. Overlay of the structures of conformer with rank 32 (ranked after the PBE-D3BJ/def2-SVP optimized energies obtained from the conformation analysis, see “Methods” in the main text) optimized in gas-phase at the PBE-D3BJ/def2-TZVPPD and the B3LYP-D3BJ/def2-TZVPPD levels of theory, respectively.

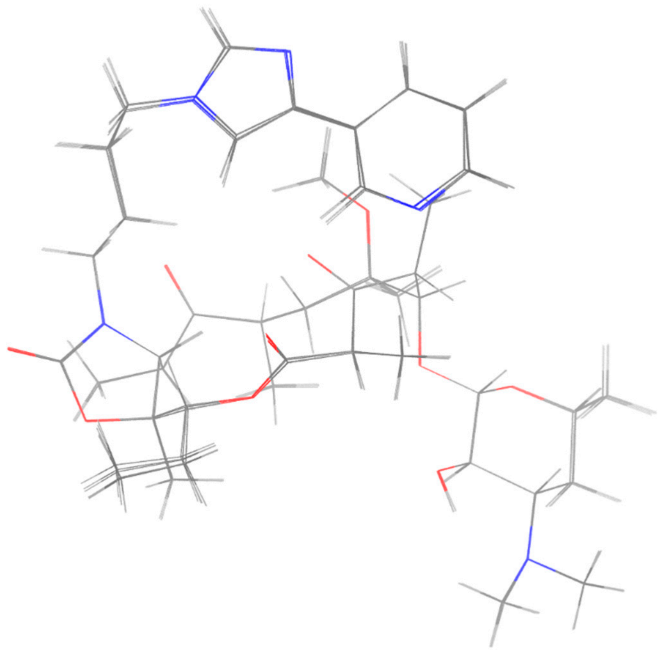

Figure S37. Overlay of the structures of conformer with rank 32 (ranked after the PBE-D3BJ/def2-SVP optimized energies obtained from the conformation analysis, see “Methods” in the main text) optimized at the PBE-D3BJ/def2-TZVPPD level of theory in gas phase and employing the COSMO model at different relative permittivities (ε_r = 1.84, 2.38, 8.93, 24.55, 80.1 – corresponding to pentane, toluene, DCM, EtOH and water, respectively).

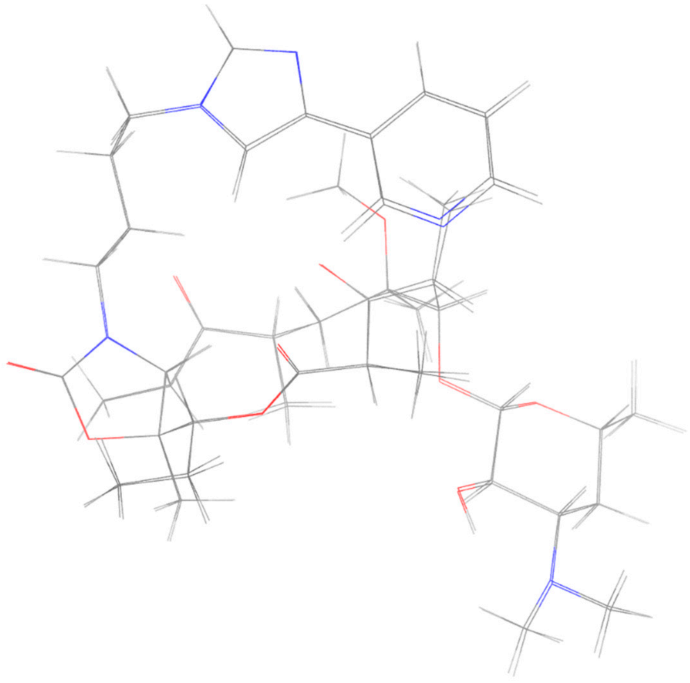

Figure S38: Overlay of the structures of conformer with rank 32 (ranked after the PBE-D3BJ/def2-SVP optimized energies obtained from the conformation analysis, see “Methods” in the main text) optimized in gas-phase at the PBE-D3BJ/def2-SVP and the PBE-D3BJ/def2-TZVPPD levels of theory, respectively.

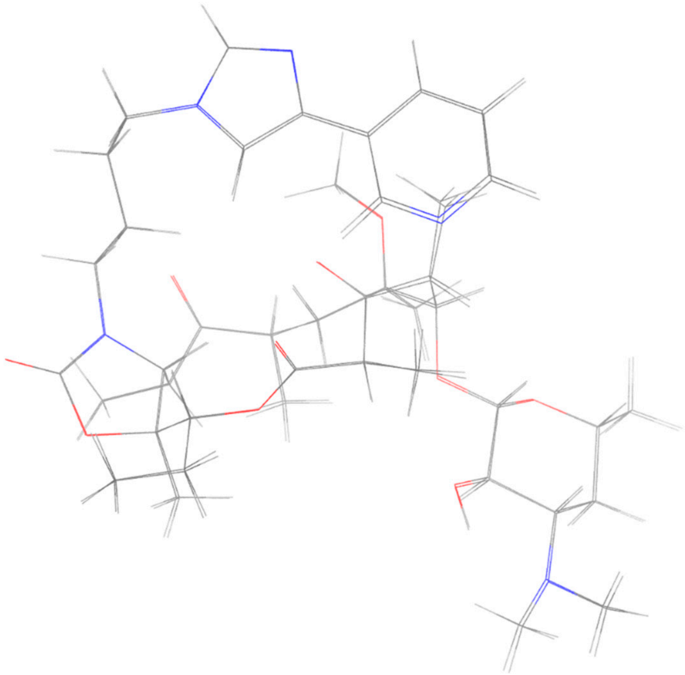

Figure S39: Overlay of the structures of conformer with rank 32 (ranked after the B3LYP-D3BJ/def2-SVP optimized energies obtained from the conformation analysis, see “Methods” in the main text) optimized in gas-phase at the B3LYP-D3BJ/def2-SVP and the PBE-D3BJ/def2-TZVPPD levels of theory, respectively.

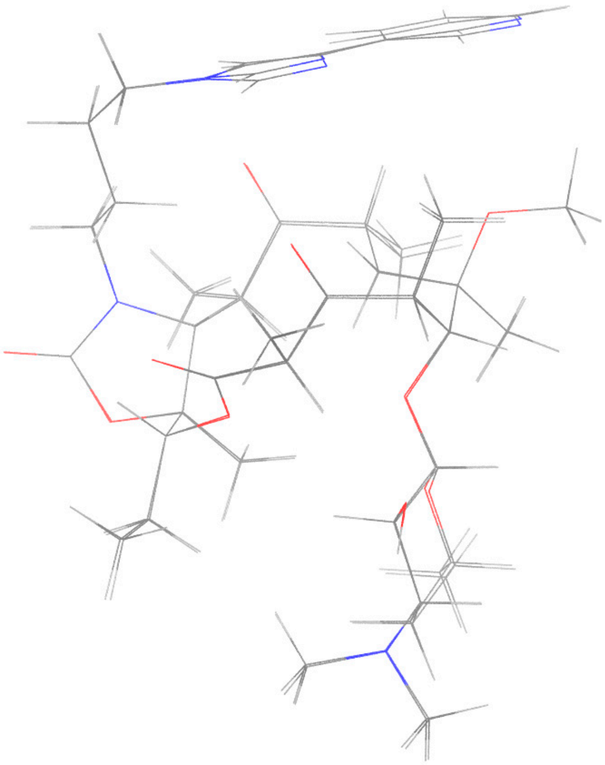

Figure S40. Overlay of the conformation depicted in Figure 4a optimized PBE-D3BJ/def2-SVP in gas-phase at employing the COSMO model for water (ε_r = 80.1, respectively).

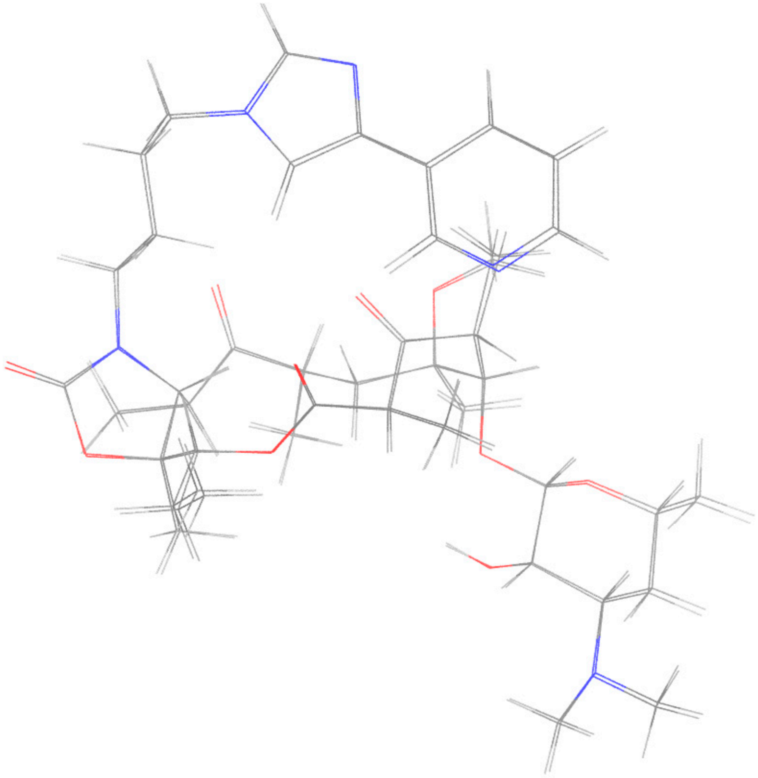

Figure S41. Overlay of the conformation depicted in Figure 4a optimized PBE-D3BJ/def2-SVP in gas-phase at employing the COSMO model for water (ε_r = 80.1, respectively).
